# Intratumoral microbiota-derived S1P sensitizes the combination therapy of capecitabine and PD-1 inhibitors

**DOI:** 10.1016/j.isci.2025.114202

**Published:** 2025-11-22

**Authors:** Chen-Shu Dai, Tian-Tian Qi, Hui-Ling Shang, Ri-Hua Xie, Hao Liu, Zhen-Ming Liu, Yi-Min Cui, Yu-Hang Zhang

**Affiliations:** 1 Institute of Clinical Pharmacology, Peking University First Hospital, Beijing 100191, China; 2 Department of Pharmacy, Wenzhou Medical University, Wenzhou 325035, China; 3 Department of Pharmacy, Xuzhou Rehabilitation Hospital, Xuzhou 221010, China; 4 Department of Nursing, Affiliated Foshan Maternity & Child Healthcare Hospital, Southern Medical University, Foshan 528100, China; 5 State Key Laboratory of Natural and Biomimetic Drugs, School of Pharmaceutical Sciences, Peking University, Beijing 100191, China

**Keywords:** Immunology, Biological sciences, Microbiome, Cancer

## Abstract

Clinical responses of colorectal cancer (CRC) treatments vary considerably due to the heterogeneity of tumor microenvironment (TME), where intratumoral microbiota may reshape the unique inflammation imprints. However, its complex mechanistic underpinnings remain incompletely elucidated. Herein, we sought to delineate the critical role of intratumoral microbiota in potentiating combination therapeutics against CRC. By comparing germ-free (GF) and specific pathogen-free (SPF) mouse models of 33 potential CRC treatments, we screened out capecitabine-MIH4 (anti-PD-1 antibody) combination regimen significantly augmented by intratumoral microbiota in tumor regression. The enrichment of enterotoxigenic *Bacteroides fragilis* induced by Capecitabine-MIH4 was concomitant with elevated microbial sphingosine-1-phosphate, which further up-regulated tumoral PD-L1 expression by enhancing histone deacetylation at the *CD274* locus. This activation ultimately led to effector memory *CD8*^*+*^ T cell expansion and exhausted T cell subset reduction within TME. To conclude, these findings highlight microbial sphingolipids as potential predictive biomarkers for strategies of targeting intratumoral microbiota in CRC management.

## Highlights

• Intratumoral microbiota promoted the treatment of Capecitabine-PD-1 inhibitor in CRC
• Capecitabine-PD-1 inhibitor combination stimulated intratumoral ETBF to generate S1P
• Activated microbial SphK2 expanded effector memory *CD8*^*+*^ T cells subsets in TME

## Graphical abstract

**Figure:**
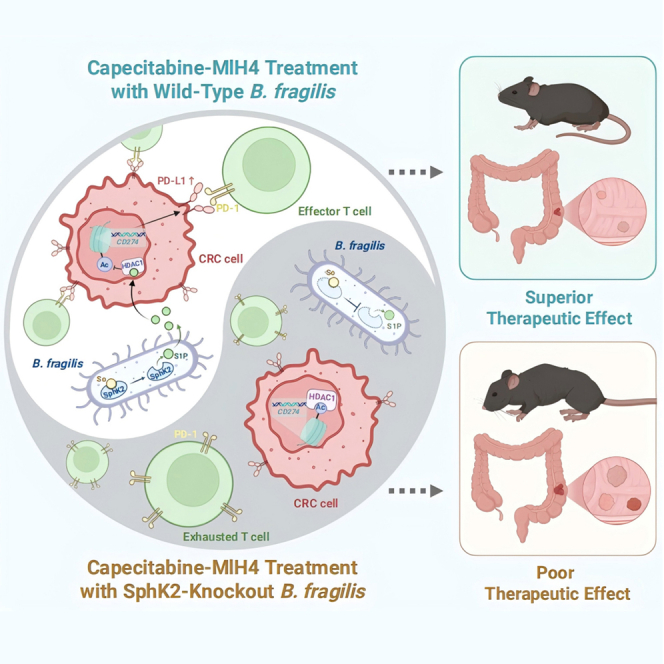

## Introduction

Colorectal cancer (CRC) has been reckoned as one of the most common malignant tumor types worldwide, with the incidence and mortality rates increasing annually.^1^^,^^2^ As research into its molecular mechanisms and therapeutic strategies deepened, an ever-growing number of combination regimens, including chemotherapy, targeted therapies, and immune checkpoint inhibitors, have been introduced into clinical practice for CRC patients.^3^^,^^4^^,^^5^ But owing to individual factors and tumor heterogeneity, the same regimen often demonstrated markedly different clinical benefits across CRC patients, underscoring the urgent need to identify more effective and broadly adaptable combination therapies.^6^^,^^7^ In recent years, the role of intratumoral microbiome has garnered increasing attention in the onset, progression, and treatment of CRC. Mounting evidence suggests that the composition and diversity of intratumoral microbiota not only influence the tumor immune microenvironment and disease progression but also closely correlated with drug efficacy and adverse reactions.^8^^,^^9^^,^^10^^,^^11^^,^^12^ Accordingly, effectively incorporating intratumoral microbiota into the assessment of therapeutic efficacy necessitates systematic investigation.

To better elucidate how the intratumoral microbiome mediates these treatments, we developed a screening platform for various CRC therapies. By comparing the tumor number and tumor volume between germ-free (GF) versus specific pathogen-free (SPF) mice, we evaluated the efficacy differences for all potential combination approaches. Among the therapies we tested, four regimens indicated the most significant discrepancies in therapeutic efficacy, including the combined use of capecitabine and MIH4 (murine programmed death receptor-1 [PD-1] monoclonal antibody). Capecitabine is a fluoropyrimidine pro-drug and remains the only globally approved agent that can be administered orally at home.^13^ It was synthesized in the 1990s,^14^ which received approval from the US Food and Drug Administration in 2005.^15^ As the monotherapy regimen of fluoropyrimidines, Capecitabine is the first-choice treatment for metastatic CRC (mCRC) in the first-line setting.^16^^,^^17^ Nivolumab, a highly selective humanized immunoglobulin G4 (IgG4) monoclonal antibody to inhibit the PD-1, is frequently applied in patients with mismatch repair-deficient/microsatellite instability-high mCRC.^18^^,^^19^ Although both agents displayed considerable anti-tumor effects, their combination therapy has not yet been adopted in clinical practice, whose underlying microbial mechanisms and therapeutic potentials remain to be investigated.

In this study, the overarching aim was to elucidate the complex host-microbiome interactions and intratumoral mechanisms underpinning the combined treatment of capecitabine and PD-1 inhibitors, paving avenues to improve this regimen in the colorectal environment. Identifying specific intratumoral microbial strains that undergo notable changes under the combination therapy, we conducted metabolomic analyses to differentiate microbial metabolites across treatment groups. Subsequently, we integrated epigenetic profiling to determine whether these microbial metabolites affected the downstream gene expression of CRC progression. In light of PD-1 inhibitors being immune checkpoint inhibitor, we performed single-cell sequencing to compare immunological variations among groups, comprehensively exploring the potential way to enhance therapeutic efficacy of capecitabine and PD-1 inhibitors combination therapy.

## Results

### Screening of CRC treatment regimens significantly affected by intratumoral microbiota

**Figure 1:**
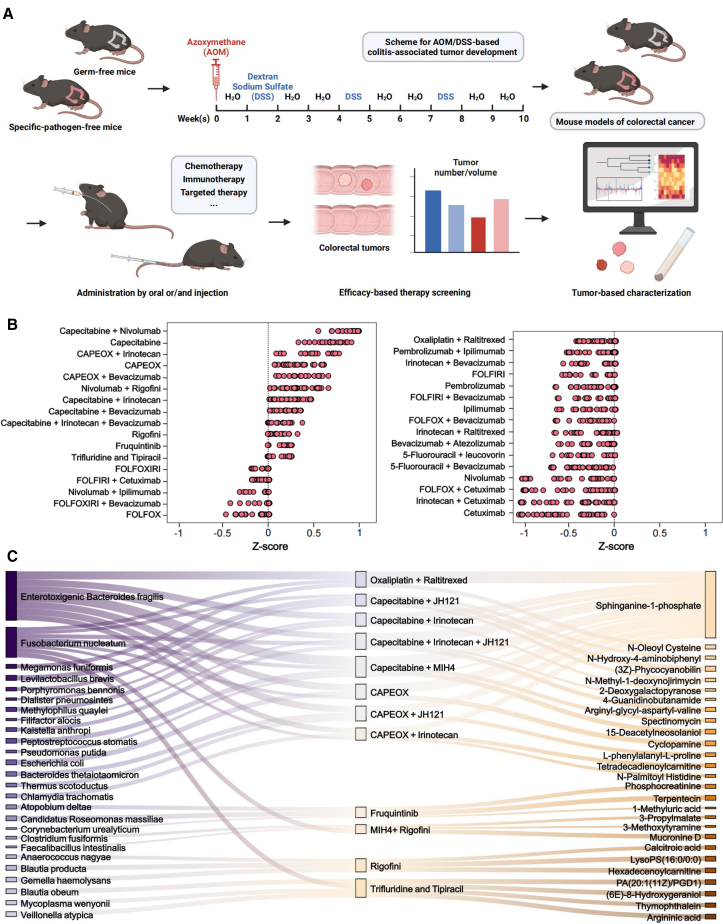
Comparative screening of anti-tumor efficacy between SPF and GF mice revealed the role of intratumoral microbiota in capecitabine-MIH4 combination (A) Schematic workflow of intratumoral microbiota-mediated anti-tumor efficacy screening platform, which was created by BioRender. Age-matched SPF and GF mice were simultaneously treated with AOM/DSS for 10 weeks, prior to 4 weeks of anti-tumor treatments. For each group of mice, therapeutic efficacy was evaluated by tumor number and volume, and CRC tissues were collected for microbiological detection. (B) *Z* factor of intratumoral microbiota effect on the given therapies in each SPF-GF mice pair, *n* = 6_SPF_ × 6_GF_ = 36. The value of *Z* factor >0.5 indicates the positive effect. (C) Microbiota-therapy-metabolite interaction network identified in this study. (Left network) Effects of intratumoral bacteria on therapeutic efficacy. Significant interactions in two independent screenings (*n* = 3 per screen) validated in a follow-up assay (*n* = 3; FDR-corrected *p* < 0.05) are shown (Spearman’s rank tests). (Right network) Differential intratumoral metabolites between SPF and GF mice of therapeutic efficacy detected by two independent screenings (Spearman’s rank tests).

To establish a screening platform for 33 different treatment regimens of CRC patients, we conducted experiments on C57BL/6 mice raised by GF and conventional (SPF) conditions. Using azoxymethane (AOM)/dextran sulfate sodium (DSS) to construct CRC model, we administered different cancer therapies (all monoclonal antibody drugs were replaced with murine antibodies) and collected CRC tissues from each group for multi-omics analysis (Figure 1A). Among 33 microbial communities validated *in vivo*, we identified four regimens with the highest *Z*-scores: capecitabine combined with MIH4, oxaliplatin combined with raltitrexed, CAPEFOX (Capecitabine combined with oxaliplatin) combined with irinotecan, CAPEFOX alone (Figure 1B), with the greatest difference observed under capecitabine-MIH4 combination therapy. Upon this screening, we initially identified the interactions between 12 treatment regimens and 26 representative microbial strains. These bacterium-therapy interaction pairs underwent two rounds of independent validations (Figure 1C). We set a false discovery rate (FDR)-adjusted *p* value <0.05 and a drug consumption rate of over 30% as the thresholds. These targeted interactions were further validated in GF mice with colonized bacteria, revealing an *in vivo* interaction network that covered all tested strains and approximately 36.36% of therapies (12 of 33 therapies).

### The enrichment of intratumoral enterotoxigenic *Bacteroides fragilis* promoted the combination treatment of capecitabine and MIH4 in CRC

**Figure 2:**
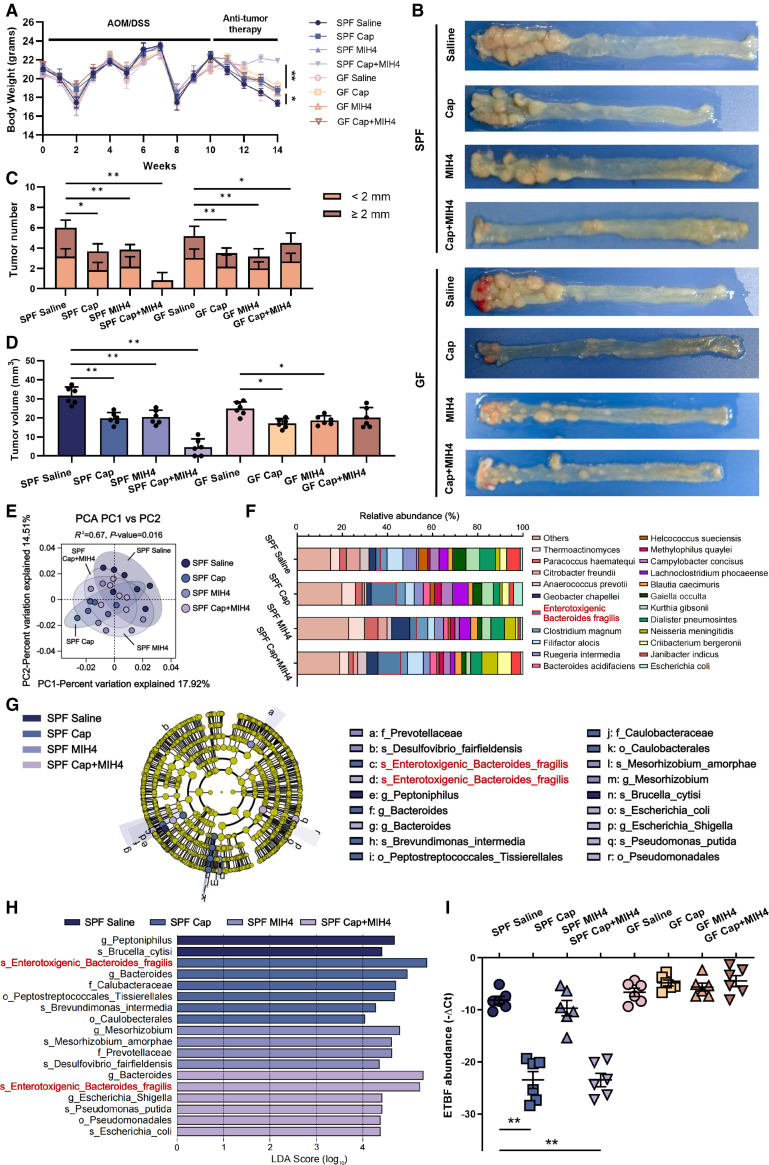
Intratumoral ETBF was enriched by the treatment of Capecitabine (A) Body weights of SPF and GF mice in each group during AOM/DSS induction and treatments (saline, capecitabine, MIH4, capecitabine-MIH4 combination). *n* = 6 mice per group (the same applies hereinafter). Data are means ± SD, ∗*p* < 0.05, ∗∗*p* < 0.01 (Student’s *t* tests). (B) Colorectal longitudinal sections of each group after 4-week anti-tumor therapies. (C and D) Colorectal tumor number (C) and volume (D) across groups. Data are means ± SD, ∗*p* < 0.05, ∗∗*p* < 0.01 (Student’s *t* tests). (E) PCA scatterplot of bacterial community β-diversity based on Bray-Curtis distances among the SPF mice groups after treatments. (F) Species taxa summary of the altered intratumoral microbiome in SPF mice treated with Capecitabine or/and MIH4. (G and H) Linear discriminant analysis (LDA) measurements to identify the significant abundance of intratumoral microbiota. Taxonomic cladogram (G) and histogram (H) were generated by LEfSe among groups. Only LDA scores >4 are shown. (I) RT-qPCR to determine the 16S rRNA segment of ETBF absolute abundance in each group. Data are means ± SD, ∗∗*p* < 0.01 (Student’s *t* tests).

To further investigate the mechanisms underlying the differential efficacy of capecitabine-MIH4 combination therapy between SPF and GF mice, we randomly assigned all SPF and GF models of CRC into four treatment groups: saline control, capecitabine alone, MIH4 alone, combination of capecitabine with MIH4. During this period, we recorded the body weight changes, diarrhea scores, and fecal blood scores for each group. Compared with GF mice, SPF mice receiving the Capecitabine-MIH4 combination maintained more stable body weights and exhibited significantly lower diarrhea and fecal blood scores (Figures 2A–2B and S1A and S1B). After sacrifice, SPF mice of the capecitabine-MIH4 combination group showcased a pronounced reduction in colorectal tumor size. By contrast, GF mice demonstrated some degrees of tumor shrinkage but remained with larger tumor volumes, suggesting a potential role of the microbial environment in treatment efficacy (*p* < 0.01; Figures 2B–2D and S1C). To further assess how different CRC treatment regimens affected intratumoral microbiota, we collected CRC tissues from SPF mice for metagenomic sequencing, obtaining an average of 45,832,565 sequencing reads per sample. Removing unannotated species shown in Mendeley database (https://data.mendeley.com/datasets/gc5c7jz5w2/1), we identified 394 microbial species classified into 157 families and 271 genera. According to α-diversity indices (ACE, Chao1, Shannon, and Simpson), no significant discrepancies were found in microbial richness or diversity among the groups (Figure S1D). Principal-component analysis (PCA) (*R*^2^ = 0.17, *p* = 0.016) and principal coordinate analysis (PCoA) (*R*^2^ = 0.71, *p* = 0.027) indicated their variations in β-diversity features (Figures 2E and S1E). Non-metric MultiDimensional Scaling (NMDS) analysis demonstrated a clustering trend across groups (stress = 0.175, Figure S1E). Subsequently, we examined the taxonomic composition of intratumoral microbiota in SPF mice (Figure 2F). Through ultra-deep metagenomic sequencing and linear discriminant analysis effect size (LEfSe) analysis, we determined that enterotoxigenic *Bacteroides fragilis* (ETBF) was prominently enriched in the capecitabine group and the capecitabine-MIH4 combination group (Figures 2G and 2H). This enrichment was further confirmed by quantitative reverse-transcription PCR (RT-qPCR) in SPF mice treated with Capecitabine alone or in combination with MIH4 (Figure 2I). Intratumoral microbial co-occurrence network illustrated the dominating microbes associated with ETBF (Figure S1F), suggesting that ETBF inevitably maintained the stability of intratumoral microbiome composition during capecitabine-MIH4 combination therapy.

### ETBF-derived sphingosine-1-phosphate upgraded intratumoral PD-L1 levels by inhibiting HDAC1 activity

**Figure 3:**
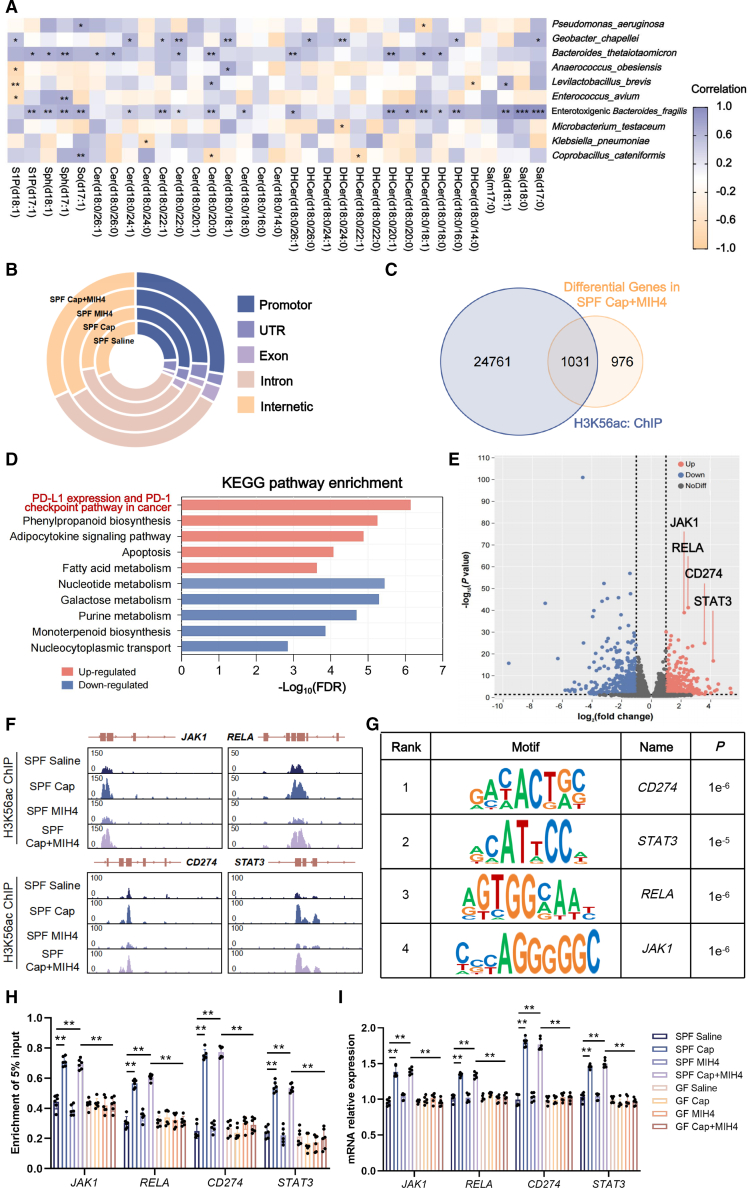
ETBF-derived S1P up-regulated the histone deacetylation of H3K56 and PD-L1 expression (A) Pearson correlation heatmap between intratumoral microbial species abundance and differential sphingolipids after capecitabine-MIH4 treatment. (B) Genomic distribution of H3K56ac peaks in CRC cells of SPF and GF mice treated with capecitabine-MIH4 combination therapy. (C) Venn diagram showing the number and overlaps of DEGs and H3K56ac-annotated genes obtained from ChIP-seq results. (D) The top five up-/down-regulated KEGG pathway enrichment in SPF mice treated with capecitabine-MIH4 combination versus saline. (E) Volcanic plot showed the differentially expressed genes of CRC tissues between SPF and GF mice treated with Capecitabine-MIH4 combination. (F) ChIP-seq profiles show the ChIP-seq signal (*y* axis, reads per million) for H3K56ac and S1P at genomic loci of *JAK1*, *RELA*, *CD274*, and *STAT3*. (G) DNA motif analysis in ChIP-seq peaks of H3K56ac showing the significant enrichment of *CD274* motif (hypergeometric test). (H) ChIP qPCR analysis of H3K56ac occupancy at genomic loci of *JAK1*, *RELA*, *CD274*, and *STAT3* in CRC tissues of SPF or GF mice treated with saline, capecitabine, MIH4, or their combination. Data are presented relative to input and shown as means ± SD, ∗∗*p* < 0.01 (Student’s *t* tests). (I) RT-qPCR of *JAK1*, *RELA*, *CD274*, and *STAT3* in CRC tissues of SPF or GF mice treated with saline, Capecitabine, MIH4, or their combination. Data are presented relative to input and shown as means ± SD, ∗∗*p* < 0.01 (Student’s *t* tests).

Given the potential mechanism underlying the efficacy of intratumoral microbiota metabolism on the synergistic effect of capecitabine and MIH4, we sought to elucidate the metabolic alterations of inter-cellular micro-ecology between GF and SPF mice. Metabolome datasets of the volcanic plots showcased higher sphingosine-1-phosphate (S1P) among all substantially discrepant intratumoral metabolites triggered by the combination treatment of capecitabine-MIH4 in SPF mice, as compared with SPF mice treated with saline as well as GF mice treated with capecitabine-MIH4 combination (Figure S2A). Neither Capecitabine alone nor MIH4 alone had obviously affected intratumoral microbiota-derived S1P for both SPF and GF mice (Figure S2B). KEGG enrichment of these multiple differences was mainly concentrated on sphingolipid (SL) metabolism (9.52%, Figure S2C). By employing ultra-performance liquid chromatography-coupled time-of-flight mass spectrometry profiling, we further quantified the significantly increased intratumoral S1P in SPF mice treated with Capecitabine and MIH4 compared with SPF mice of the saline group or GF mice (Figure S2D). To further discriminate the origins of microbial metabolites, all closely associated bacteria-metabolite pairs were identified at class, order, family, genus, and species levels (*p* < 0.05). Correlation analyses confirmed a significant association between ETBF and elevated intratumoral S1P levels in the capecitabine-MIH4 combination treatment group (Figure S2E). Pearson correlation coefficient between major classes of microbial SLs and candidate taxa (at the species level) also manifested the specifically negative correlation of ETBF with intratumoral microbiota-derived S1P(d17:1) or S1P(d18:1), respectively (*p* < 0.001, Figure 3A). Phylogenetically, the majority of bacterial species cannot produce SLs. But *Bacteroides*, one of the most predominant commensal genera in intratumoral microbiota, can produce and provide a source of ceramides with both odd (C17:0) and even (C18:0) numbers of hydrocarbons.^20^^,^^21^ The biosynthesis of intratumoral S1P originated from the phosphorylation of sphingosine via the enzymes microbial sphingosine kinase 1/2 (SphK1/2, Figure S3A). To identify key enzymes of modulating downstream S1P levels in response to pharmacological agents, we biosynthesized mutant bacterial strains with inactivated SphK1/2 (BFΔSphK1 and BFΔSphK2, Figure S3B). Then we co-cultured SW620, SW480, HT-29, HCT116, or HCT-15 cells with wild-type ETBF (BFWT) or BFΔSphK1/2 cells within the transwell system and added ceramide to determine whether ETBF*-*derived S1P can be transferred to mammalian hosts to interfere with drug efficacy (Figure S3C). High-resolution MS2 spectra also revealed the significantly decreased S1P in various CRC cell lines when co-cultured with BFΔSphK2, in comparison to those co-cultured with BFWT or BFΔSphK1 (Figure S3D). These indicated that S1P catalyzed by microbial SphK2 can penetrate into CRC cells, thus improving the combination treatment of Capecitabine and MIH4.

It was previously reported that S1P serves as an endogenous inhibitor of histone deacetylase 1/2 (HDAC1/2) to regulate histone acetylation modification in *p21* and *c-fos* promoter region.^22^ However, the specific modification sites by the combination treatment of capecitabine-MIH4 and whether it affects the transcription of other target genes are still unclear. Extracting total histone from their CRC tissues, we determined the main differences in histone modification sites of SPF mice treated with Capecitabine or Capecitabine-MIH4 combination as H3K56 acetylation (H3K56ac), comparing with other groups (Figures S4A and S4B). To determine whether the combination treatment of capecitabine and MIH4 could prompt epigenetic changes within CRC tissues, RNA sequencing was applied for SPF and GF mice treated with capecitabine-MIH4 combination, which resulted into a total of 2,007 differentially expressed genes (DEGs), including 1,029 up-regulated and 978 down-regulated genes (*p* < 0.05, Figure S4C). Then chromatin immunoprecipitation sequencing (ChIP-seq) analysis for H3K56ac was thus performed in CRC tissues of SPF mice and GF mice. There were 19,353 (28.5%) gained and 15,946 (23.5%) lost H3K56ac peaks in SPF mice compared with GF mice, but their distributions of H3K56ac across the genome were similar. As compared with GF mice, the binding sites of H3K56ac and downstream target genes in SPF mice were more concentrated in the promoter region and exon region (Figure 3B). Comprehensive transcriptomic profiling of H3K56ac demonstrated that over half of the DEGs (1,031 genes, 51.4%) exhibited either H3K56ac gain or loss in the combination group (Figure 3C). Kyoto Encyclopedia of Genes and Genomes (KEGG) pathway analysis further indicated significant enrichment of up-regulated genes in tumoral PD-L1 expression and PD-1 checkpoint pathway, implying that the Capecitabine-MIH4 combination potentiates PD-L1 synthesis machinery (Figure 3D). Notably, the combined regimen induces up-regulation of 59 genes related with PD-L1 synthesis, including *JAK1*, *RELA*, *CD247*, and *STAT3* (Figure 3E). ChIP-seq profiling also revealed increased H3K56ac peaks at these genes (Figure 3F). We subsequently sought to identify transcription factors involved in chromatin remodeling by Capecitabine-MIH4 combination treatment in SPF mice. Motif analysis of H3K56ac peaks in SPF mice using HOMER revealed that *CD274* motif was significantly enriched at H3K56ac peaks (Figure 3G). Considering that MIH4 efficacy mainly correlates with intratumoral PD-L1 expression, these findings indicate that Capecitabine in combination therapy promoted microbial S1P production and synergistically enhanced the efficacy of MIH4 by up-regulating PD-L1 synthesis of CRC tissues (Figures 3H and 3I).

### Intratumoral SphK2 activity of ETBF modulated the anti-tumor efficacy of Capecitabine-MIH4 combination therapy

**Figure 4:**
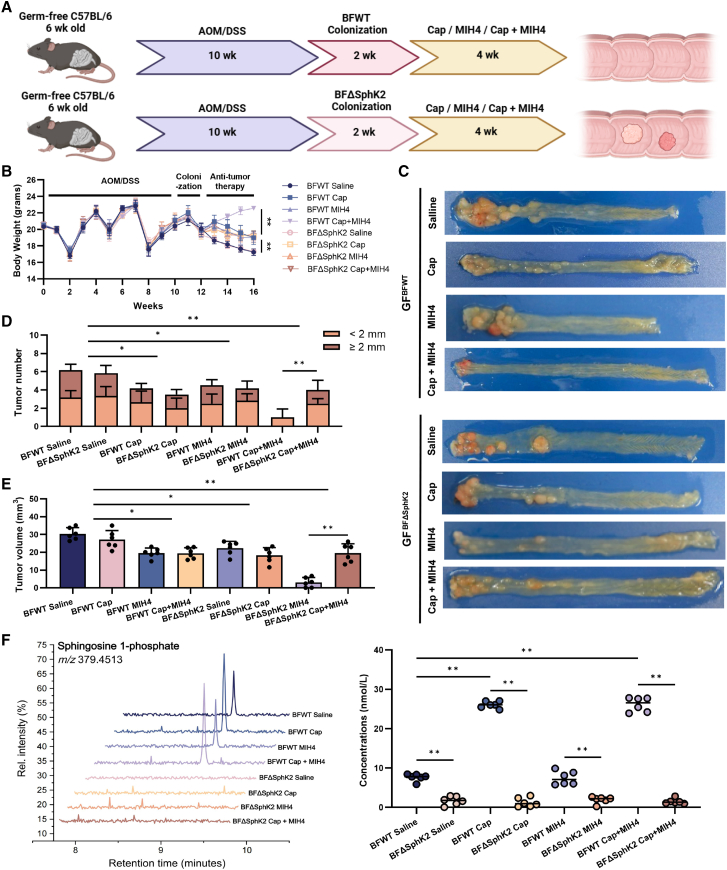
S1P production capacity of colonized ETBF affected efficacy of capecitabine-MIH4 combination (A) Experimental setting: age-matched GF mice were simultaneously induced with 10 weeks of AOM/DSS before 2 weeks of BFWT or BFΔSphK2 colonization, followed by the treatment of saline, capecitabine, MIH4, or their combination for 4 weeks (B) Body weight of each group during 16 weeks of AOM/DSS induction and 2 weeks of BFWT/BFΔSphK2 colonization followed by treatment with anti-tumor therapies for 4 weeks *n* = 6 mice per group, ∗∗*p* < 0.01 (Student’s *t* tests). (C) Colorectal longitudinal sections of each group. (D and E) The number (D) and volume (E) of tumors in the colon and rectum were measured in GF^BFWT^ and GF^BFΔSphK2^ mice after the above 16 weeks of CRC model construction and treatments. (F) Liquid chromatography-tandem mass spectrometry to quantify intratumoral S1P across the groups, ∗∗*p* < 0.01 (Student’s *t* tests).

To tightly monitor microbial SphK2-catalyzed S1P production, we mono-colonized 6-week-old GF mice with BFWT or BFΔSphK2 strain (Figures S5A), prior to the above treatments (Figure 4A). As shown in Figure 4B, both Capecitabine and MIH4 obviously affected the body weights of GF mice colonized with BFWT (GF^BFWT^) and BFΔSphK2-colonized GF mice (GF^BFΔSphK2^). The combination of Capecitabine and MIH4 induced greater weight gain in GF^BFWT^ mice than those of GF^BFΔSphK2^ mice (*p <* 0.01). Capecitabine outperformed MIH4 in ameliorating diarrhea, bloody feces, and tumor proliferation of both GF^BFWT^ and GF^BFΔSphK2^ mice, while symptoms of GF^BFWT^ mice were superior to GF^BFΔSphK2^ mice when treated with Capecitabine-MIH4 combination therapy (*p <* 0.01, Figures S5B, S5C, and S4C). GF^BFΔSphK2^ mice treated by Capecitabine/MIH4 manifested no distinct difference in colonic length and tumor number and volume, compared with GF^BFWT^ mice (Figures 4D and 4E and S5D). Nevertheless, GF^BFWT^ mice treated with Capecitabine-MIH4 combination therapy showed statistically longer colon length and less tumor number and volume than GF^BFΔSphK2^ mice (*p <* 0.05; Figures 4D and 4E and S5D). High-resolution MS2 spectra quantified the dramatic increase of intratumoral S1P in GF^BFWT^ mice treated with capecitabine alone or combination therapy, as compared with the saline controls (*p <* 0.01, Figure 4F).

### Single-cell dynamics and cross-tissue landscape of CRC patients treated with capecitabine-nivolumab combination therapy

**Figure 5:**
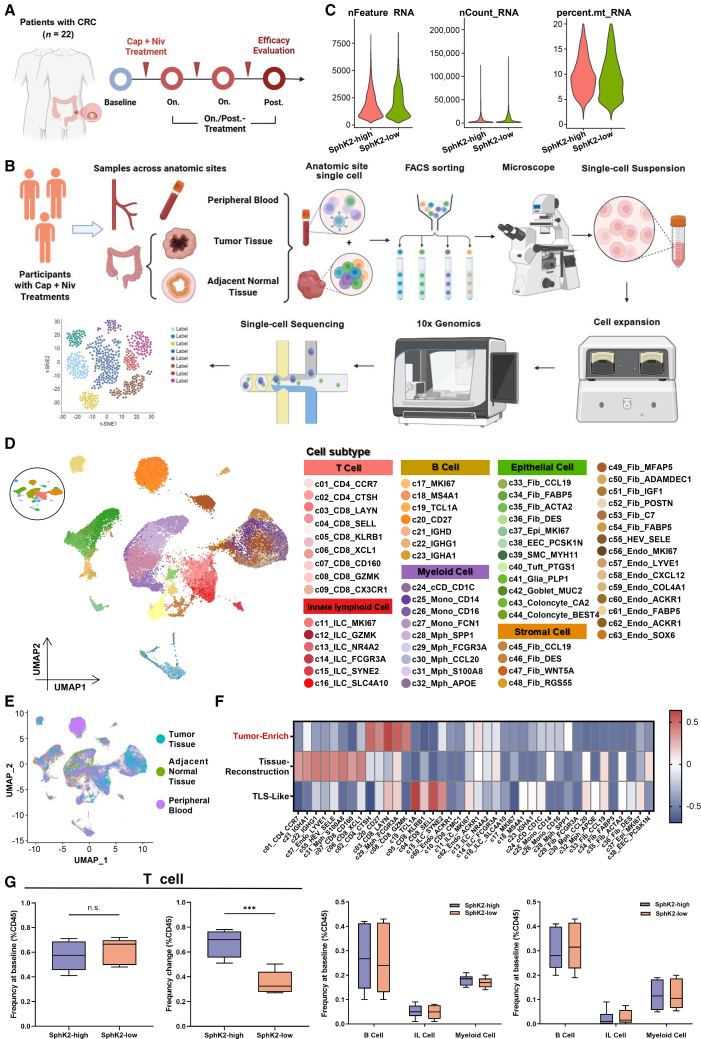
Dynamic single-cell landscape of CRC patients during Capecitabine-Nivolumab treatment (A) Dynamic tracking of 22 CRC patients undergoing Capecitabine-Nivolumab treatment. (B) Workflow of sample collection and analysis of single-cell RNA-seq. (C) Quality control of single-cell assay for transposase-accessible chromatin using sequencing (scATAC-seq) data (left). Distribution of accessible chromatin regions per nucleus (nFeature; cutoff: >500; middle). Log_10_-scaled distribution of total fragments per nucleus (nCount; right). Proportion of mitochondrial reads (percent.mt; exclusion threshold: <20%). (D) Uniform manifold approximation and projection (UMAP) plot of cell clusters. Cell types were identified by the regulation activity matrix and then visualized by UMAP. Cells were colored according to annotated cell types. (E) UMAP plots showing the anatomic site. (F) Tissue preference of each cluster estimated by observed-to-expected ratio (Robs/exp). The Robs/exp was *Z* score transformed. (G) Boxplots showing the relative expression levels of immune cells in 22 CRC patients. *p* value was calculated using Student’s *t* test, ∗∗∗*p* < 0.01.

To elucidate the dynamic effects of Capecitabine combined with PD-1 inhibitors treatment, we applied single-cell RNA sequencing (scRNA-seq) to longitudinally track CRC patients receiving capecitabine and nivolumab, a representative PD-1 inhibitor, combination therapy throughout the treatment course. Based on the strict inclusion criteria, 22 CRC patients were enrolled with each subject undergoing baseline (pre-treatment) sampling followed by one or more post-treatment samplings after initial drug administration, including the follow-up surgery when necessary (Figure 5A). To comprehensively map the local and systemic dynamics, CRC tissues obtained from colonoscopic or surgical biopsies were paired with peripheral blood and adjacent normal tissue samples, which were all processed immediately for matched scRNA-seq (Figure 5B). We divided all subjects into SphK2-high activity group and SphK2-low activity group based on their microbial SphK2 activities and examined 918,367 cells for further analysis according to the parameter settings (nFeature-RNA >500 and percentage. mt < 20; Figure 5C). The transcriptomes of 815,265 high-quality single cells were selected after stringent quality control and filtration. Unsupervised clustering combined with classical marker gene annotation identified six major cell populations: T cells, B cells, innate lymphoid cells (ILCs), myeloid cells, stromal cells, and epithelial cells (Figure 5D). Separated sub-clustering on each major compartment resulted in 63 fine-grained cell subsets, which were all characterized by their distinctive expression profile as well as distribution patterns across the blood, adjacent normal tissues, and tumor tissues (Figure 5E). To statistically quantify tissue enrichment of each sub-population, we measured the ratio of observed to expected cell numbers (Robs/exp). By comparing organizational distribution preferences, we found that helper T cells (c02-CD4_CTSH), memory B cells (c20uCD27), exhausting T cells (c03_CD8_LAYN), macrophages (c29_Sph_SCGR3A), and effector memory T cells (c08_CD8_GZMK) were mainly enriched in CRC tissues (Figure 5F). For each major and fine-grained cell type, the baseline cellular abundance and cellular dynamics (characterized as the difference between the post-treatment and baseline abundance) were calculated. Intratumorally, we observed disparate response associations of the four major immune cell types. Notably, B cells, ILCs, and myeloid cells demonstrated no significant inter-group difference from baseline to post-treatment. After combination therapy, the SphK2-high activity group manifested significantly elevated T cell infiltration when compared to the SphK2-low group (Figure 5G), suggesting enhanced T cell-mediated anti-tumor immunity in the former group. Metagenome profiles of CRC patients also indicated similar α-diversity, β-diversity, and dominant colonies between the two groups (Figures S5E–S5H). By collecting their CRC tissues, we determined that SphK2-high patients had markedly higher intratumoral S1P and PD-L1 concentrations (*p <* 0.01, Figures S5I and S5J). It thus provides us with critical insights into the potential role of microbial SphK2 in modulating immunotherapy response and offers perspectives for optimizing personalized strategies of capecitabine and PD-1 inhibitors.

### Associations of exhausted T cells with the combination efficacy of capecitabine and nivolumab

**Figure 6:**
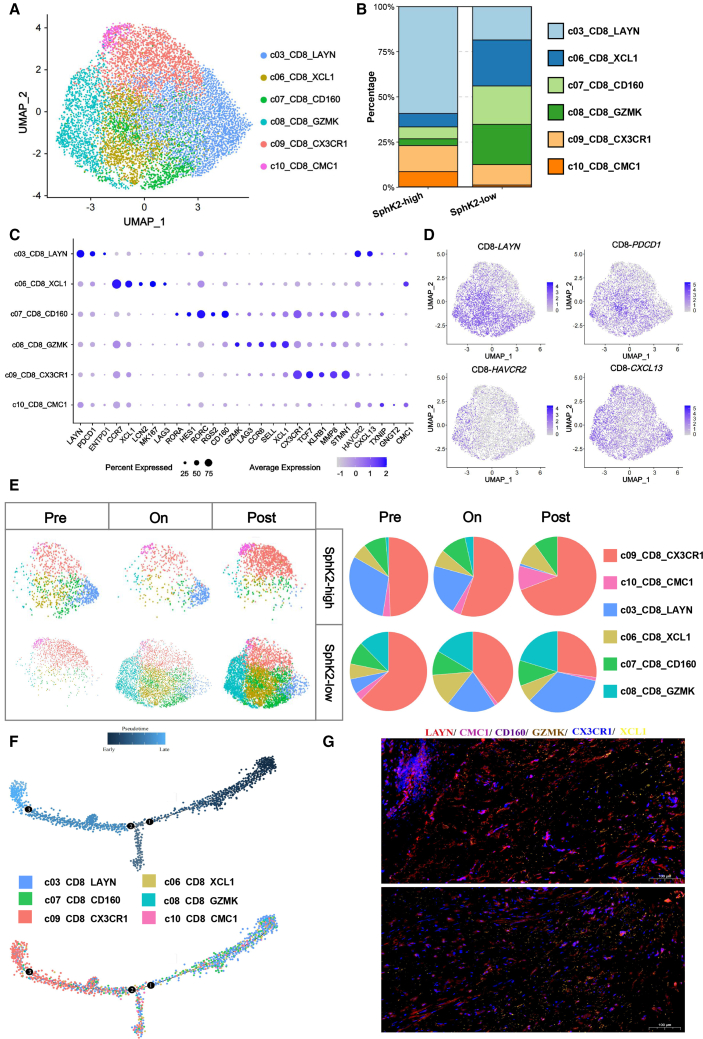
Tumor-reactive-like *CD8*^+^ T cells under Capecitabine-Nivolumab treatment (A) Sub-clustering of *CD8*^*+*^ T cell clusters, colored and labeled by subtypes. (B) Clustering of *CD8*^+^ T cell clusters in groups with different microbial SphK2 activities. (C) Bubble heatmap showing the expression of representative signature genes of *CD8*^+^ T subtypes. Both the color and size indicate scaled mean expression across cell subtypes for each gene. (D) UMAP plots showing the expression of representative genes. (E) UMAP plots showing the dynamics of *CD8*^+^ T cell clusters following the capecitabine-nivolumab treatment (left). Pie charts showing the *CD8*^+^ T subtype composition of representative changes across different treatment stages (right). (F) Differentiation trajectory of *CD8*^+^ T cells in CRC, with each color coded for pseudotime and clusters. (G) Representative multiplex immunohistochemical (mIHC) staining of T cells in SphK2-high dataset; colored staining as annotated. Scale bars, 20 μm.

Considering the aforementioned findings that microbial SphK2 mediated capecitabine-PD-1 inhibitor combination efficacy, coupled with the observed dynamic patterns of diverse immune cell populations, we subsequently focused on the T cell compartment and sub-clustering T cells into their established phenotypes using a graph-based clustering algorithm (Figure 6A). Unsupervised clustering analysis of all T cell sub-clusters revealed that exhausted T cells (Tex) constituted the predominantly altered sub-population (Figure 6B). Each population was defined by known classical signature genes. Given that Tex cells exhibited significant expression of *PDCD1* and other exhaustion-related genes, including *LAYN*, *HAVCR2*, and *CXCL13*, we thus directed our focus on *CD8*^+^ Tex (Figures 6C and 6D). Longitudinal tracking of Tex cell sub-population dynamics throughout the treatment course further substantiated this distinct phenotypic conversion between SphK2-high and SphK2-low cohorts, which suggested that Capecitabine combined with Nivolumab may reverse T cell exhaustion by inhibiting *LAYN* expression. To decipher the pertinent effects of Capecitabine combined with Nivolumab blockade among deferentially responsive groups, we performed comprehensive profiling of Tex cell sub-populations to delineate distinct patterns between SphK2-high and SphK2-low cohorts. Notably, post-treatment analysis revealed the substantial accumulation of terminally Tex cells (c03_CD8_LAYN) in CRC tissues from SphK2-low patients. Intriguingly, the abundance of terminally exhausted and proliferating Tex cell populations was predominantly replaced by effector memory T cells (c08_CD8_GZMK) following immunotherapy for microbial SphK2-low patients (Figure 6E). In pseudotime analysis, we found that CD8_LAYN are localized at the start of the Monocle trajectories in the SphK2-high group and CD8_ CX3CR1 are localized at the end (Figure 6F). According to these findings, CD8_LAYN could convert into CD8_CX3CR1 (Figure 6G). Gene set enrichment analysis (GO-BP and Hallmark) identified the significant up-regulation of immune response pathways in SphK2-high activity group following Capecitabine and Nivolumab treatment (Figures S6A and S6B). Building upon the observed dynamic interplay between *LAYN*^*+*^ exhausted *CD8*^*+*^ T cells and functionally competent T cell populations, we comprehensively evaluated their combined prognostic significance in our patients' follow-up. Notably, treatment with Capecitabine and Nivolumab demonstrated superior clinical outcomes in microbial SphK2-high patients, with significantly improved disease-free survival ([DFS] 70 months versus 130 months; hazard ratio [HR], 0.81; *p* < 0.05) and overall survival (OS: 180 months versus 230 months; HR, 0.69; *p* < 0.05) compared to microbial SphK2-low counterparts (Figure S6C). Predicting which patients will benefit from preoperative capecitabine and nivolumab, is the crucial objective to improve patient outcomes and to provide organ-preserving options for operable CRC. Based on the above indicators treated with capecitabine and nivolumab, the presence of microbial SphK2 activity and the relative abundance of intratumoral ETBF were negatively correlated with tumor mutational burden (TMB) and carcinoembryonic antigen (CEA) levels (Figure S6D).

## Discussion

Intratumoral microorganisms primarily originate from intestines, oral cavities, and adjacent tissues, where intestinal mucosal damage and blood circulation facilitate their colonization within tumors.^10^^,^^23^^,^^24^ Predominant microorganisms enriched in CRC include *Fusobacterium nucleatum,* polyketide synthase (pks)^+^ *Escherichia coli*, and *B. fragilis.*^25^^,^^26^^,^^27^ These intratumoral microbiota promote CRC progression by modulating intestinal epithelial cells, neoplastic cells, and tumor microenvironment. Mechanistically, their oncogenic effects involve critical biological processes such as genotoxic DNA damage induction, dysregulation of apoptotic pathways, and promotion of epithelial-mesenchymal transition.^28^^,^^29^ The intricate interplay between intratumoral microbiota and host anti-tumor immunity provides a nuanced perspective for developing combination therapies. In this study, we have conducted a comparative evaluation of the anti-tumor efficacy between GF and SPF mice models under capecitabine and MIH4 combination therapy. It manifested significantly superior tumor inhibition in SPF mice as compared to their GF counterparts, indicating a pivotal regulatory role of intratumoral microbiota in therapeutic responsiveness. This observation aligns with the established literature documenting that GF or antibiotic-treated mice exhibited impaired responses to immune-checkpoint blockade therapy, whereas *Bacteroides* supplementation effectively restores therapeutic efficacy.^30^ Through intratumoral microbiota composition and metabolic profiles across treatment groups, we identified intratumoral microbiota-derived S1P as the critical metabolite in mediating differential therapeutic outcomes. Of note, elevated intratumoral S1P derived from ETBF directly interacts with HDAC1 in CRC cells, suppressing its deacetylase activity and enhancing H3K56ac.^22^ Through ChIP-seq and ChIP-qPCR analyses, we further demonstrated that intratumoral S1P-mediated H3K56ac exhibits significant enrichment within the *CD274* gene locus, leading to the markedly up-regulated PD-L1 transcription in CRC tissues of SPF mice subjected to combination therapy. These findings collectively indicate that microbial S1P enhanced CRC tissue dependency on the PD-L1 pathway by inhibiting HDAC1 and elevating histone acetylation at the PD-L1 genomic region, ultimately synergizing with MIH4 to potentiate therapeutic efficacy.

Previous studies have revealed that deficiency of *Bacteroides*-derived SLs induces intestinal inflammation and disrupted host lipid metabolism in GF mice,^21^ underscoring the pivotal role of microbiota-synthesizing S1P to regulate host immunoregulation processes. Our single-cell sequencing analyses further elucidated the relationship between microbial SphK2 activity and dynamics of tumor-infiltrating T cell lineages. Among CRC patients with high microbial SphK2 activity, combination therapy induced a significant expansion of effector memory *CD8*^*+*^ T cells within the tumor microenvironment, accompanied by a relative reduction in Tex cell subsets. In contrast, CRC patients with low microbial SphK2 activity retained a higher proportion of terminally Tex cells. Gene set enrichment analysis demonstrated pronounced enrichment of immune activation signaling in the SphK2-high activity cohort post-treatment. Furthermore, our data suggest that microbial SphK2 may regulate the equilibrium between T cell exhaustion and memory differentiation through analogous mechanisms, thereby dynamically modulating immune response intensity. Integrated analysis of clinical cohort data revealed that CRC patients with elevated microbial SphK2 activity exhibited superior clinical outcomes following Capecitabine plus Nivolumab therapy, including significantly prolonged DFS and OS. These findings not only provide the biomarker for clinical stratification but also indicate microbial SL metabolites as potential predictive tools for assessing patient responsiveness to immunotherapeutic interventions.

Taken together, our systematic investigation elucidates that intratumoral S1P produced by ETBF modulates immune checkpoint expression through an epigenetic regulatory pathway, potentiating the therapeutic efficacy of Capecitabine and PD-1 inhibitor combination therapy. This discovery significantly enhances our comprehension of intratumoral microbiota involvement in oncotherapy and immune regulation. Future research should prioritize delineating mechanistic roles of other pivotal microorganisms and their metabolites in cancer treatment paradigms, and translating these findings into clinically actionable intervention strategies would enable more personalized and precision therapeutic approaches for CRC and other malignancies.

### Limitations of the study

There are certain limitations to this study. The translation of microbial SphK2 activity into clinically applicable biomarkers requires standardized quantification methodologies. Future studies should prioritize developing mass spectrometry-based assays to measure SphK2-derived S1P in tumor biopsies or non-invasive liquid biopsies. Combining this with single-cell meta-transcriptomics could help resolve intratumoral microbial heterogeneity. Such integrated approaches may facilitate patient stratification for microbiota-targeted adjuvant therapies. Furthermore, the study only investigated the effects of SphK2 derived from ETBF on Capecitabine-PD-1 inhibitor combination therapy in CRC patients. It is essential to comparatively profile SphK2 activity across different bacterial species and correlate enzymatic heterogeneity with *in vivo* immunomodulatory potency. This would clarify whether microbial SphK2 functionality is strain-specific or broadly conserved, thereby refining its applicability into the clinical practice.

## Resource availability

### Lead contact

Further information and requests for resources and reagents should be directed to the lead contact, Yuhang Zhang (yuhang@pkufh.cn).

### Materials availability

This study did not generate new unique reagents.

### Data and code availability

The 16S rRNA sequencing and single-cell RNA sequencing data supporting the results in this study are deposited in Mendeley Data (DOI: https://doi.org/10.17632/gc5c7jz5w2.1) (https://data.mendeley.com/datasets/gc5c7jz5w2/1). The minimum dataset for main figures and supplementary figures that support the findings of this study are openly available in Figshare (DOI: https://doi.org/10.6084/m9) (https://doi.org/10.6084/m9.figshare.30359296). Source data are provided with this paper.

## Acknowledgments

This project was supported by 10.13039/100014718National Natural Science Foundation of China (82204515, 82574496, and W2523107), Beijing Municipal 10.13039/100007219Natural Science Foundation (7232262), National High Level Hospital Clinical Research Funding (Research Achievement Transformation Project of 10.13039/100017415Peking University First Hospital) (2022RT04, 2022SF04, 2022CR118, and 4803021), and 10.13039/501100019005Young Elite Scientists Sponsorship Program by 10.13039/100010097CAST and 10.13039/501100020437BAST (YESS20240292 and 2023BJ204679).

## Author contributions

Y.-H.Z. and Y.-M.C. have conceived the project. H.-L.S. and R.-H.X. collected clinical samples and pathological analysis. C.-S.D. and T.-T.Q. have implemented experiments of pharmaceutical analysis. Z.-M.L. and H.L. performed statistical analysis for multi-omics data. C.-S.D. and Y.-H.Z. wrote the manuscript, which was edited by all authors.

## Declaration of interests

The authors declare no competing interests.

## STAR★Methods

### Key resources table

**Table:** 

REAGENT or RESOURCE	SOURCE	IDENTIFIER
**Antibodies**		

CD279 (PD-1) Monoclonal Antibody (MIH4)	Fisher Scientific	Cat#14-9969-82
VEGF Monoclonal Antibody (JH121)	Fisher Scientific	Cat#MA5-13182
EGFR Monoclonal Antibody (H11)	Fisher Scientific	Cat#MA5-13070
CD152 (CTLA-4) Mono- clonal Antibody (UC10-4B9)	Fisher Scientific	N/A
Anti-TCF21 antibody	Fisher Scientific	Cat# PA5-116012
Anti-IgG antibody	Fisher Scientific	Cat# MA5-49460
Anti-CD235a antibody	BioLegend	Cat# 349113
Purified Rat Anti-Mouse CD16/CD32	BD Biosciences	Cat# 567021
Anti-7AAD antibody	BD Biosciences	Cat# 559925
Anti-CD235a antibody	BioLegend	Cat# 116703

**Bacterial strains**		

*Escherichia coli* BL21(DE3) Chemically competent cells	MilliporeSigma	Cat#CMC0014
*Bacteroides fragilis*	ATCC	Cat#25285

**Experimental models: Cell lines**		

SW620	ATCC	Cat#CCL-227
SW480	ATCC	Cat#CCL-227
HT-29	ATCC	Cat#HTB-38
HCT116	ATCC	Cat#CCL-247EMT
HCT-15	ATCC	Cat#CCL-225

**Chemicals, peptides, and recombinant proteins**		

Semipurified diet	Fisher Scientific	ICN96029610
Azoxymethane	Fisher Scientific	Cat# 451657
Dextran sulfate sodium	Fisher Scientific	Cat# A7631
Methanol	Fisher Scientific	Cat# A20006
Tetrodotoxin	Fisher Scientific	Cat# HB1035
TRIzol	Fisher Scientific	Cat# P1675
Formaldehyde	Fisher Scientific	Cat# 552233
Glycine	Fisher Scientific	Cat# V2002
Protein A magnetic beads	Fisher Scientific	Cat# 88845
Dynabeads™ Oligo (dT) 25	Fisher Scientific	Cat# 61005
SuperScript™ II Reverse Transcriptase	Fisher Scientific	Cat# 18064071
Gentamicin	Sigma-Aldrich	Cat# G1397
Erythromycin	Sigma-Aldrich	Cat# E5389
RPMI-1640 medium	Fisher Scientific	Cat# 11875093
HISTOPAQUE-1077	Sigma-Aldrich	Cat# 10771
ACK lysing buffer	Fisher Scientific	Cat# A1049201
Paraformaldehyde Solution, 4% in PBS	Fisher Scientific	Cat# J19943.K2

**Critical commercial assays**		

Magnesium RNA Fragmentation Module	New England Biolabs	Cat# e6150
SuperScript™ II Reverse Transcriptase	Invitrogen	Cat# 18064022
SimpleChIP® Enzymatic Chromatin IP Kit	Cell Signaling Technology	Cat# 9003
NEXTflex® ChIP-Seq kit	BioScientific	Cat# NOVA-5143-02
gentleMACS™ Dissociator	Miltenyi Biotec	Cat# 130-093-235
ReverTraAce qPCR PT Kit	Toyobo	Cat# FSQ-101
PowerUp™SYBR™Green Master Mix Kit	Fisher Scientific	Cat# A25741

**Deposited data**		

Metagenome data for mice colorectal cancer (CRC) tissues	This paper	released upon publication
Non-targeted metabolome data for mice CRC tissues	This paper	released upon publication
H3K5ac ChIP-seq fastq files for mice CRC tissues	This paper	released upon publication
Single cell RNA sequencing data for CRC tissues of patients	This paper	released upon publication

**Experimental models: Organisms/strains**		

Mouse: C57BL/6J	GemPharmatech	N/A
Mouse: Germ-free C57BL/6J	GemPharmatech	N/A

**Oligonucleotides**		

Primers for *27F* F: GTTTGATCCTGGCTCAG	Integrated DNA Technologies	N/A
Primers for *1492R* R: CGGCTACCTTGTTACGAC	Integrated DNA Technologies	N/A
Primers for *B. fragilis* F: TCRGGAAGAAAGCTTGCT R: ACACGTATCCAACCTGCCCTTTACTCG	Integrated DNA Technologies	N/A
Primers for *Gapdh* F: TGACGTGCCGCCTGGAGAAA R: AGTGTAGCCCAAGATGCCCTTCAG	Integrated DNA Technologies	N/A

**Software and algorithms**		

R v4.4.2	MathSoft	https://www.r-project.org/
Python v3.4.1	Python Software Foundation	https://www.python.org/
StringTie v3.0.0	Johns Hopkins University	https://ccb.jhu.edu/software/stringtie/
Trimmomatic v0.40	N/A	https://www.usadellab.org/cms/?page=trimmomatic
MACS2 v2.0	PyPI	https://github.com/taoliu/MACS
EasyGO	N/A	https://bioinformatics.cau.edu.cn/easygo
FlowJo v10.10	BD Biosciences	https://www.flowjo.com/
ImageJ	NIH	https://imagej.nih.gov/ij/
GraphPad Prism v9.0	GraphPad Prism	https://www.graphpad.com/

**Other**		

16S rRNA sequencing and single-cell RNA sequencing data	Mendeley Data	https://doi.org/10.17632/gc5c7jz5w2.1
Minimum dataset for Figures and Supplementary Figures	Figshare	https://doi.org/10.6084/m9.figshare.30359296.v1
Agilent 1290 Infinity UPLC system	Agilent Technologies	N/A
Sciex TripleTOF 6600 mass spectrometer	SCIEX	N/A
GTxResolve Premier BEH Amide Column	Waters	Cat# 186011250
NanoDrop ND-1000 spectrophotometer	Fisher Scientific	N/A
Agilent 2100 Bioanalyzer	Agilent Technologies	N/A
Novaseq™ 6000	illumina	N/A
LSRFortessa™ Cell Analyzer	BD Biosciences	Cat# 10004D
FLOQSwabs® sterile swab	Copan	Cat# 56380

### Experimental model and study participant details

#### Animal experiments

All animal procedures were performed in accordance with protocols approved by Peking University First Hospital Experimental Animal Center (Ethical No. 2022-23142). C57BL/6J germ-free (GF) and specific pathogen-free (SPF) mice were maintained under controlled conditions (temperature: 20-22°C; humidity: 40-60%) with a 12-h light-dark cycle and *ad libitum* access to food and water. After one-week acclimatization period, mice were assigned to experimental groups. Colorectal cancer (CRC) mice model was established by the single intraperitoneal injection of azoxymethane (AOM; 10 mg/kg), followed by three cycles of dextran sulfate sodium (DSS) administration (2% DSS in drinking water for 1 week, alternating with normal drinking water for 2 weeks *per* cycle).

For the mice experiments in Figures 1 and 2, GF and SPF mice were treated with varieties of CRC therapies for 4 weeks (*n* = 6 for each group). At the end of week 14, all mice were euthanized by cervical dislocation. The colorectum was dissected from cecum to anus, longitudinally incised, photographed, and examined for tumor enumeration. The number, volume of colorectal tumors was recorded. The *Z*_i_ is calculated using the following equation:

$$Z_{i}=1\;-\;3\times \frac{\sigma_{p}+\sigma_{n}}{\mu_{p}\;-\;\mu_{n}}$$

wherein, p represents the positive group (SPF mice), n represents the negative group (GF mice), σ is standard deviation of tumor volume in each group, and μ is the average tumor volume of each group. CRC tissues were collected for further analysis (refer to ‘Metagenomic sequencing and analysis' and ‘Metabolome analysis of intratumoral microbiota' sections). Subsequently, quantitative PCR (qPCR) was applied to validate microbial abundances, and LC-MS was used to quantify metabolites of the initial screening results. After false discovery rate (FDR) correction, statistical significance was assessed using Wilcoxon’s rank sum test with a *P*-value cut-off of 0.05. For *in vivo* experiments depicted in Figure 4, all CRC-induced mice were divided into four treatment groups: Saline group, Capecitabine group, MIH4 group, and Capecitabine-MIH4 combination group. Prior to 2-week drug administration, mice were colonized either BFWT or BFΔSphK2 strains *via* dietary supplementation for 4 weeks.

#### Human sample collecting and processing of scRNA-Seq

CRC tissue biopsies were collected from the enrolled participants, along with 1 mL of peripheral blood during each colonoscopy procedure. The Ethics Committees of Peking University First Hospital approved the study protocols (Ethical number: 2022-166), which is publicly accessible through the Chinese Clinical Trial Registry (ChiCTR2400088785; http://www.chictr.org.cn/historyversionpub.html?regno=ChiCTR2400088785). Written informed consents were obtained from all participants in this study. For CRC tissue samples, excised biopsies were immediately preserved in tissue preservation solution and transported on ice. After two washes, tissues were minced into 1-2 mm^3^ fragments and enzymatically dissociated using the gentleMACS Tumor Dissociation Kit in RPMI-1640 medium supplemented with 10% fetal bovine serum (FBS) for 60 minutes at 37 °C on a rotor. Dissociated cells were filtered through 100 μm SmartStrainers (pluriSelect) into RPMI-1640 medium containing 10% FBS, centrifuged at 400 *g* for 5 minutes, and pelleted. The supernatant was discarded, and cells were resuspended in 1 mL of erythrocyte lysis buffer and incubated on ice for 1 minute, followed by quenching with RPMI-1640 medium. After centrifugation for 5 minutes at 400 *g*, the cell pellet was resuspended in sorting buffer (PBS supplemented with 1% FBS). Peripheral blood samples were collected in EDTA anticoagulant tubes and cryopreserved until processing. After thawing to room temperature and thorough mixing, peripheral blood mononuclear cells (PBMCs) were isolated using HISTOPAQUE-1077 by centrifugation at 400 *g* for 30 minutes. The immune cell layer was carefully transferred to a new tube, washed twice with PBS, and subjected to erythrocyte lysis as described above. The isolated immune cells were finally resuspended in sorting buffer.

### Method details

#### Metagenomic sequencing and analysis

High-quality sequences from each sample were clustered into operational taxonomic units (OTUs) using UPARSE (v7.0.1001) at 97% sequence similarity. Taxonomic annotation was carried out against the SILVA138 database, followed by statistical analysis across taxonomic ranks (phylum to species). Alpha diversity indices (ACE, Chao1, Shannon and Simpson) were used to measure community richness and diversity of microbes. For beta diversity analysis, uniFrac distances were calculated in QIIME (v1.7.0), with sample relationships visualized *via* UPGMA (Unweighted Pair Group Method with Arithmetic Mean) clustering trees. Principal component analysis (PCA) and principal coordinates analysis (PCoA) were implemented in R software to reveal sample-to-sample variation, using the FactoMineR package for dimensionality and ggplot2 package for visualization. Potential biomarkers were identified through Linear Discriminant Analysis Effect Size (LEfSe), focusing on statistically significant (*P* < 0.05) and biologically relevant (LDA score ≥ 4) features to identify taxa with notable inter-group differences.

#### Real-time qPCR

RT-qPCR was performed to determine the relative abundance of intratumoral bacteria. Total RNA from CRC tissues was isolated using TRIzol reagent and reversely transcribed into cDNA with the ReverTraAce qPCR PT Kit. qPCR was performed in triplicate using the PowerUp™SYBR™Green Master Mix Kit with target-specific primers. For absolute abundance quantification of ETBF, a 16S rRNA fragment was amplified using universal primers 27F/1492R. The resulting PCR product was serially diluted to generate a standard curve, followed by amplification with bacteria-specific qPCR primers (Table S2). Data are reported as -ΔCt 16S rRNA gene copies of fecal content.

#### Metabolome analysis of intratumoral microbiota

Metabolomic profiling was conducted on 50 mg CRC tissue per mouse. For untargeted analyses, samples were initially homogenized in 80% cold methanol, followed by centrifugation at 21,500 *g* for 15 minutes at 4 °C to collected supernatants for LC-MS. LC-MS was performed on an Agilent 1290 Infinity UPLC system coupled to a Sciex TripleTOF 6600 mass spectrometer. Chromatographic separation was carried out using a Waters BEH Amide column (2.1 mm × 100 mm, 1.7 μm). Raw MS data were converted to mzXML format *via* ProteoWizard, then processed in R using XCMS package (v3.2) to generate a data matrix encompassing retention time (RT), mass-to-charge ratio (*m/z*) and peak intensity. The CAMERA package (v16.4) annotated peaks, with metabolites identification against MS2 spectral database. Processed data were analyzed using MetaboAnalyst package (v4.0) to quantify metabolite abundances. Metabolites displaying significant alterations were identified by Wilcox rank-sum tests, with FDR-adjusted *P* < 0.05, and associations between differentially altered bacteria and metabolites were assessed *via* partial spearman correlation, visualized through heat-maps generated with the ComplexHeatmap R package.

#### RNA sequencing analysis

Total RNA was extracted from samples by TRIzol reagent following the manufacturer’s manual. The RNA concentration and integrity were assessed using a NanoDrop ND-1000 spectrophotometer and a Agilent 2100 Bioanalyzer. Then poly (A) RNA was purified from 1μg of total RNA through two rounds of purification with Dynabeads Oligo (dT), which was fragmented using Magnesium RNA Fragmentation Module at 94 °C for 5-7 minutes. The fragmented RNA pieces were reversely transcribed into cDNA by SuperScript™ II Reverse Transcriptase and sequenced with Illumina Novaseq™ 6000. Then transcript quantification was performed applying StringTie (v2.1.4) and differential expression analysis conducted with edgeR (v3.21). The differentially expressed mRNAs and genes were picked with log_2_(fold change) >1 or log_2_(fold change) <-1 and statistical significance (FDR-adjusted *P* value < 0.05).

#### Chromatin immunoprecipitation (ChIP) and ChIP-seq

ChIP assay was carried out using SimpleChIP® Enzymatic Chromatin IP Kit according to manufacturer protocols. In brief, TPCs overexpressing TCF21 (TPC_NM_^TCF21^) were washed twice with cold PBS, treated with 1% formaldehyde for 10 minutes at room temperature to cross-link proteins and DNA, followed by quenching with 125 mM glycine. After two cold PBS washes, all cells were lysed on ice, chromatin was fragmented *via* sonicationinto to 150-900 bp fragments. For immunoprecepitaion, an aliquot of 20 μL of chromatin was reserved as input control, and another 100 μL was incubated overnight at 4 °C with 10 μg of anti-TCF21 antibody. An anti-IgG antibody served as the negative control. A total of 10 μg of anti-TCF21 antibody was added to each immunoprecipitation reaction before incubated overnight at 4 °C. The samples were then incubated with 30 μL of protein A beads for 2 hours. After reversing the cross-links and purifying the DNA, the immunoprecipitated DNA was quantified by quantitative PCR. For ChIP-Seq, libraries were prepared using NEXTflex® ChIP-Seq kit and sequenced (150-bp paired-end) on the illumina Xten platform. For data analysis, low-quality reads were quality-trimmed using Trimmomatic, and peak were called with MACS2 (v2.2.7.1) using default settings (bandwidth 300 bp; model fold 5, 50; *q* value 0.05). Peaks were assigned to the gene whose transcription start site (TSS) was closest to the peak summit.

#### Microbial strains and gene-editing strategies

ETBF strains isolated from CRC tissues of enrolled participants were identified by 16S rRNA gene sequence against the NCBI reference database. For sphingosine kinase 2 (SphK2) overexpression, the full-length microbial *SphK2* open reading frame (ORF) was cloned into pET28a vector with 6 × His tag at N-terminal end adopting standard molecular cloning procedures. Microbial *SphK2* was induced in *E. coli Rosetta* (DE3), which were cultured to an OD600 of 0.6 at 37 °C. For SphK2 knockout, an internal fragment (290 bp) of the *SphK2* gene from ETBF was inserted into the pGERM suicide vector containing selective markers of *E. coli* (*bla*). The construct was subsequently electroporated into the conjugative donor *E. coli* S17 strain. Then *E. coli* S17 as donor bacteria and ETBF as receptor bacteria were co-cultured under aerobic conditions, which were transferred to BHI medium agar plates for mutant selection by gentamicin (200 μg/mL) and erythromycin (25 μg/mL) at 37 °C for 24 hours. Primers targeting junction regions between pGERM and *SphK2* genes were utilized to pick resistant colonies for qPCR identification. Both wild-type and *SphK2*-depleting ETBF were grown at 37 °C in LB medium for 24 h. Bacterial pellets were centrifuged for 10 minutes at 8,000 *g* and 4 °C, and resuspended with oxygen-free PBS to obtain bacteria for oral administration. Colonization efficacy of the two strains in mice was confirmed by strain-specific PCR (2% agarose gel electrophoresis) and quantified using a GelDoc™ CR + Imaging System (Figure S5A).

#### Single-cell sources and data generation

To obtain high-quality cell pools prior to subsequent library construction, single-cell suspensions isolated from CRC tissue samples were stained with anti-7AAD antibody and anti-CD235a antibody for fluorescence-activated cell sorting (FACS) using a BD Aria III cell sorter. Live cells were enriched by gating on *7AAD*^*-*^*CD235a*^*-*^ populations to exclude residual erythrocytes. Gated cells were sorted into 1.5 mL LoBind tubes, manually counted under a microscope, and adjusted to a concentration of 500-1,200 cells/μL. Approximately 10,000-18,000 cells were loaded into the 10 × Chromium Single Cell 5' Library and VDJ Library Construction system, followed by standardized manufacturer’s protocols. Purified libraries were sequenced on an Illumina NovaSeq platform with 150-bp paired-end reads.

For blood samples, red blood cells were lysed using ACK lysing buffer according to the manufacturer’s instructions. After washing with PBS containing 2% FBS, cells were stained with surface antigen-specific antibodies at 4 °C for 30-60 minutes. For intracellular staining, cells were fixed with 4% paraformaldehyde (PFA) at 4 °C for 1 hour and permeabilized with methanol at -20 °C overnight. Cells were then blocked with Purified Rat Anti-Mouse CD16/CD32 at room temperature for 10 minutes, followed by incubation with intracellular antigen-specific antibodies at 4 °C for 1 hour. Data were acquired using a BD LSRFortessa flow cytometer and analyzed using FlowJo software.

#### Cell differentiation trajectory

To infer the differentiation trajectory of intratumor T cells, we applied an unsupervised inference method Monocle2 to infer the pseudotime of each cell. Genes with mean expression > 0.1 were selected in the trajectory analysis, followed by dimension reduction with the DDRTree method and construction of pseudo-temporal order. Finally, the trajectories were visualized as 2D t-SNE maps.

#### Multiplex fluorescence immunohistochemistry

We performed multiplex fluorescence immunohistochemical staining according to the kit manufacturer’s instruction. After multiple rounds of repeated antigen repair, incubation of primary antibody, HRP labeling of secondary antibody and amplification of TSA fluorescence signal, a paraffin section was marked with multiple target fluorescence labeling, and finally DAPI was used to re-stain the nucleus. Spectral imaging was performed with a multi-spectral tissue imaging system, followed by Image scanning and analysis using caseviewer and Image J.

#### scRNA-seq data processing

The sequencing data were mapped to the human reference sequence (GRCh38). The raw gene expression matrix from each sample was aggregated and converted into a Seurat object *via* Seurat (v5) package in R software. Non-immune cells with > 6000 or < 200 genes or > 40% mitochondrial genes were discarded. Immune cells with > 4000 or < 200 genes or > 25% mitochondrial genes were filtered out. To further eliminate the data of doublets, we performed the scrublet pipeline for each batch of our scRNA-seq data, which were expected to exclude doublets, and the expected_doublet_rate was set at 0.05. The gene expression matrices were normalized to the total unique molecular identifiers (UMI) counts *per* cell and transformed to the natural log scale. To correct the technical and biological variations and increase the accuracy of cell type designation, we applied canonical correlation analysis implemented in Seurat to all samples before cell type identification.

Seurat workflow was employed to perform dimensionality reduction and unsupervised clustering. Initially, the top 2000 highly variable genes (HVGs) were selected using the *FindVariableFeatures* function with parameters set to *method = “vst”*. Subsequently, the *ScaleData* function was applied to regress out the effects of total UMI counts and mitochondrial gene percentage from the HVG expression matrix. PCA was conducted on the scaled HVG expression matrix *via* the *RunPCA* function of Seurat, retaining the top 30 principal components for downstream analyses. A systematic two-round unsupervised clustering strategy based on the *scanpy.tl.leiden* function was executed to resolve cellular population architecture. During the first round, clusters were annotated through canonical lineage marker expression patterns, enabling identification of major cell types including T cells, B cells, ILCs, myeloid cells, stromal cells and epithelial cells. Clusters exhibiting high co-expression of two or more lineage markers were classified as doublets and excluded for subsequent analyses. Iterative refinement of major cell type annotation and doublet removal was performed to ensure purity across all primary cellular compartments. For each major cell type, a second round of unsupervised clustering was implemented using the aforementioned protocol to delineate fine-grained sub-types, with analyses exclusively restricted to the expression matrix of cells within the targeted cellular compartment. For each cellular sub-type, we assessed its distribution patterns across blood, adjacent normal tissue and tumor tissue by calculating the observed-to-expected (*Ro/e*) ratio. Pearson residuals, derived from the chi-square test, were further employed to quantify distributional differences between tumor and adjacent normal tissues. For this analysis, cells exclusively from these two tissue types were included as input for the test function.

### Quantification and statistical analysis

#### Statistical analysis

At least three independent experiments were carried out, and their outcomes are shown as mean ± standard deviation (*SD*). GraphPad Prism was employed for statistical analyses. Error bars in both scatter plots and bar graphs indicate *SD*. Comparisons between groups were conducted utilizing Student’s *t*-test, Mann-Whitney U-test, one-way analysis of variance (ANOVA), Bonferroni and Scheffe post-test for continuous variables. Spearman’s Rank Correlation was used to evaluate associations between variables.
