# Supplementary material for: Intratumoral microbiota-derived S1P sensitizes the combination therapy of capecitabine and PD-1 inhibitors
Source: iScience. 2025 Nov 22;28(12):114202. doi: 10.1016/j.isci.2025.114202 (PMC12723371; doi:10.1016/j.isci.2025.114202)
Supplement: Document S1. Figures S1–S6 and Tables S1–S2 [file mmc1.pdf]

iScience, Volume *28*

Supplemental information

Intratumoral microbiota-derived S1P sensitizes the combination therapy of capecitabine and PD-1 inhibitors

Chen-Shu Dai, Tian-Tian Qi, Hui-Ling Shang, Ri-Hua Xie, Hao Liu, Zhen-Ming Liu, Yi-Min Cui, and Yu-Hang Zhang

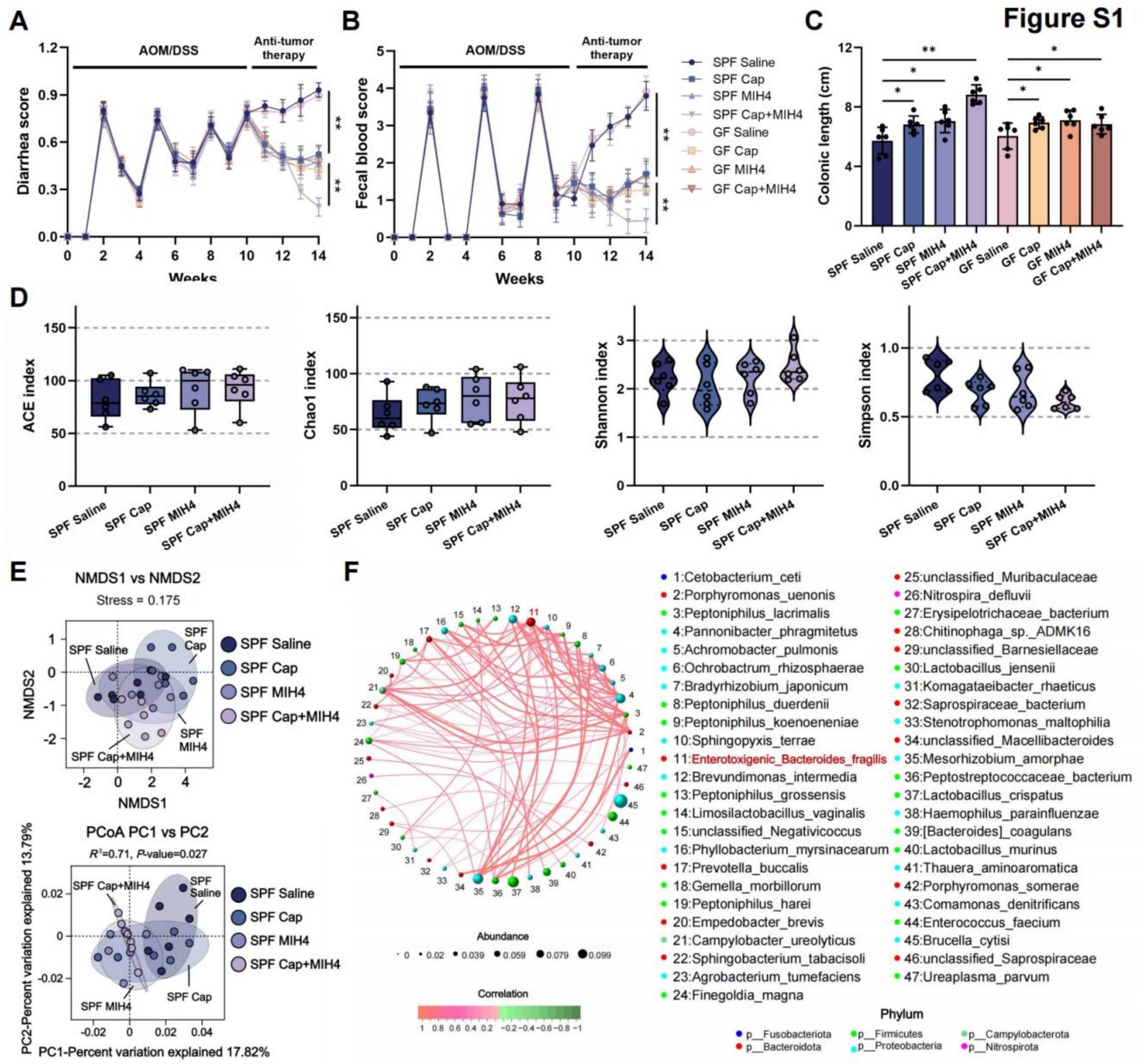

Figure S1. Intratumoral microbiota is indispensable in enhancing the efficacy of Capecitabine-MIH4 combination therapy for colorectal cancer, related to Figure 2. (A and B) Diarrhea score (A) and fecal blood score (B) of SPF and GF mice in each group were evaluated during AOM/DSS induction and drug treatments. $n = 6$ mice per group (the same applies hereinafter). Data are means ± *SD*, $^{**}P < 0.01$ (Student's *t*-tests). (C) Colon lengths were measured across groups by daily treatment of saline, Capecitabine, MIH4 or Capecitabine-MIH4 combination. Data are means ± *SD*, $^{*}P < 0.05$, $^{**}P < 0.01$ (Student's *t*-tests). (D) α-diversity of intratumoral microbiota among SPF mice treated with Saline, Capecitabine, MIH4 or Capecitabine-MIH4 combination, as indicated by ACE, Chao1, Shannon and Simpson indexes. (E) β-diversity

of intratumoral microbiota across groups, as indicated by the scores of PCoA and NMDS. (F) Analysis of intratumoral microbial co-occurrence network focused on core microbial species whose average relative abundance greater than 0.1%. The size of the nodes implies the abundance of corresponding bacteria. The colored lines connecting nodes indicate the nature of correlations—pink for positive and green for negative—and the line thickness corresponds to the correlation strength (Spearman's rank correlation coefficient).

Figure S2

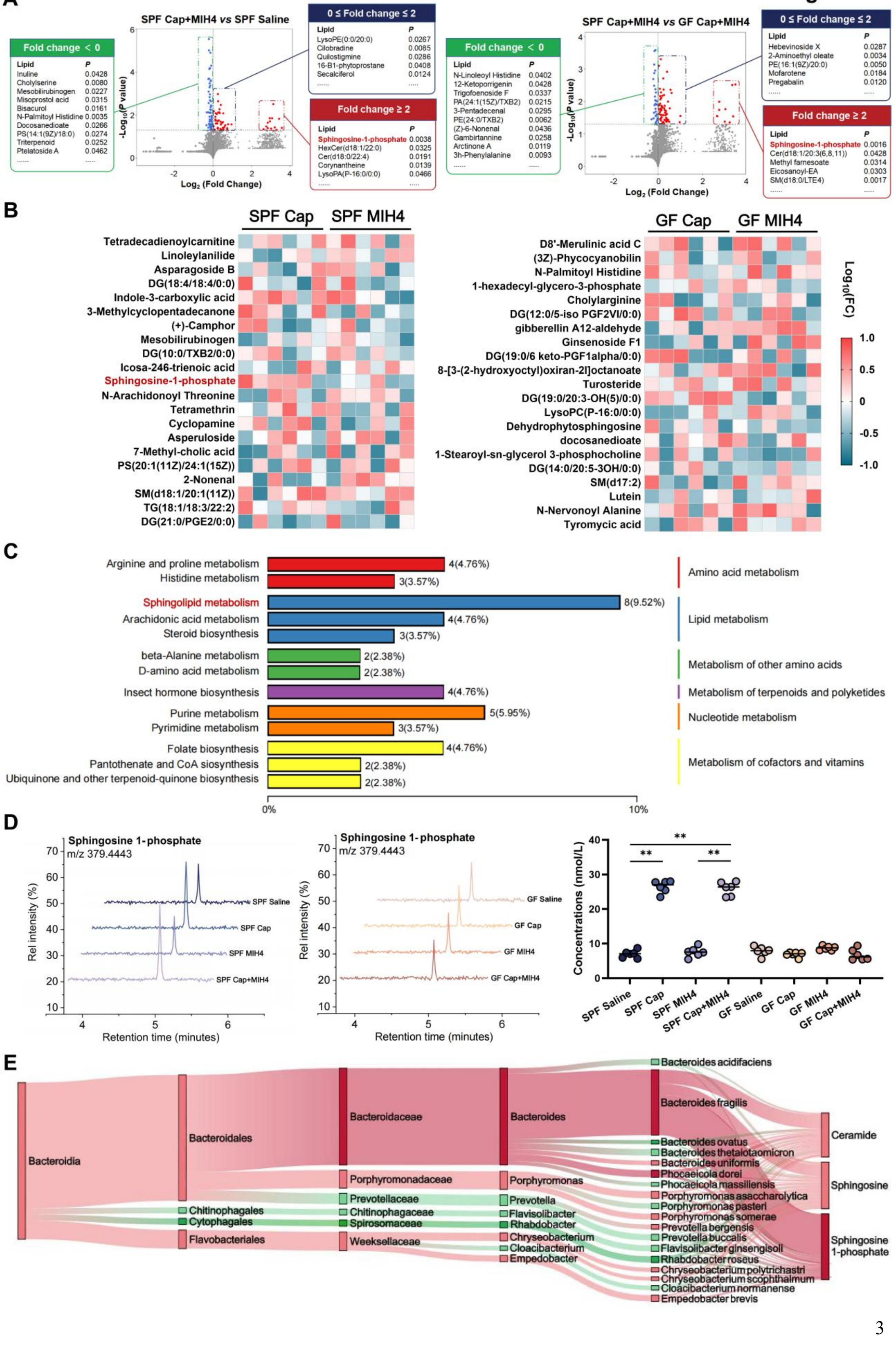
A
SPF Cap+MIH4 vs SPF Saline
SPF Cap+MIH4 vs GF Cap+MIH4
Fold change < 0
0 ≤ Fold change ≤ 2
Fold change ≥ 2
Sphingosine-1-phosphate
Log2 (Fold Change)
-Log10(P value)
B
SPF Cap
SPF MIH4
GF Cap
GF MIH4
Log10(FC)
C
Arginine and proline metabolism 4(4.76%)
Histidine metabolism 3(3.57%)
Sphingolipid metabolism 8(9.52%)
Arachidonic acid metabolism 4(4.76%)
Steroid biosynthesis 3(3.57%)
beta-Alanine metabolism 2(2.38%)
D-amino acid metabolism 2(2.38%)
Insect hormone biosynthesis 4(4.76%)
Purine metabolism 5(5.95%)
Pyrimidine metabolism 3(3.57%)
Folate biosynthesis 4(4.76%)
Pantothenate and CoA siosynthesis 2(2.38%)
Ubiquinone and other terpenoid-quinone biosynthesis 2(2.38%)
Amino acid metabolism
Lipid metabolism
Metabolism of other amino acids
Metabolism of terpenoids and polyketides
Nucleotide metabolism
Metabolism of cofactors and vitamins
D
Sphingosine 1-phosphate
m/z 379.4443
Rel intensity (%)
Retention time (minutes)
SPF Saline
SPF Cap
SPF MIH4
SPF Cap+MIH4
GF Saline
GF Cap
GF MIH4
GF Cap+MIH4
Concentrations (nmol/L)
E
Bacteroidia
Bacteroidales
Chitinophagales
Cytophagales
Flavobacteriales
Bacteroidaceae
Porphyromonadaceae
Prevotellaceae
Chitinophagaceae
Spirosomaceae
Weeksellaceae
Bacteroides
Porphyromonas
Prevotella
Flavisolibacter
Rhabdobacter
Chryseobacterium
Cloacibacterium
Empedobacter
Bacteroides acidifaciens
Bacteroides fragilis
Bacteroides ovatus
Bacteroides thetaiotaomicron
Bacteroides uniformis
Phocaeicola dorei
Phocaeicola massiliensis
Porphyromonas asaccharolytica
Porphyromonas pasteri
Porphyromonas somerae
Prevotella bergensis
Prevotella buccalis
Flavisolibacter ginsengisoli
Rhabdobacter roseus
Chryseobacterium polytrichastri
Chryseobacterium scophthalmum
Cloacibacterium normanense
Empedobacter brevis
Ceramide
Sphingosine
Sphingosine 1-phosphate

Figure S2. Capecitabine-MIH4 combination activates ETBF-derived sphingolipid metabolism, related to Figure 3. (A) Volcanic plots to illustrate intratumoral metabolites difference between Capecitabine-MIH4-treated SPF mice and saline-treated SPF mice (left panel)/ Capecitabine-MIH4-treated GF mice (right panel). Dots corresponding to significant lipids (P < 0.05, Student's *t*-tests) were colored, in which lipids with increased fold change were colored red, and sphingolipids with decreased fold change pertained to green. (B) Heat-map analysis of top 21 metabolites in intratumoral metabolites between Capecitabine-treated, MIH4-treated SPF mice (left panel) and GF mice (right panel), respectively. Each column represents one independent sample, and each row represents one metabolite. The color indicates the relative abundance of metabolites in each group. (C) Histogram presentation of the KEGG pathway. A total of 87 differentiated functional pathways were successfully annotated and grouped into 6 functional categories. *P* values were determined using two-sided Fisher's exact tests with Benjamini-Hochberg correction for multiple testing. (D) LC-MS/MS to quantify S1P metabolites in each group. Data are means ± *SD*, $^{**}P < 0.01$ (Student's *t*-tests). (E) Sankey network with microbial Ceramide, Sphingosine and S1P for Capecitabine-MIH4-treated SPF mice. The red/green color of the nodes indicates the statistically significant up/down-regulation (Student's *t*-tests). The dark red/green bands indicate the positive/negative correlations with metabolites of statistically significance, $P < 0.05$ (Spearman's rank tests).

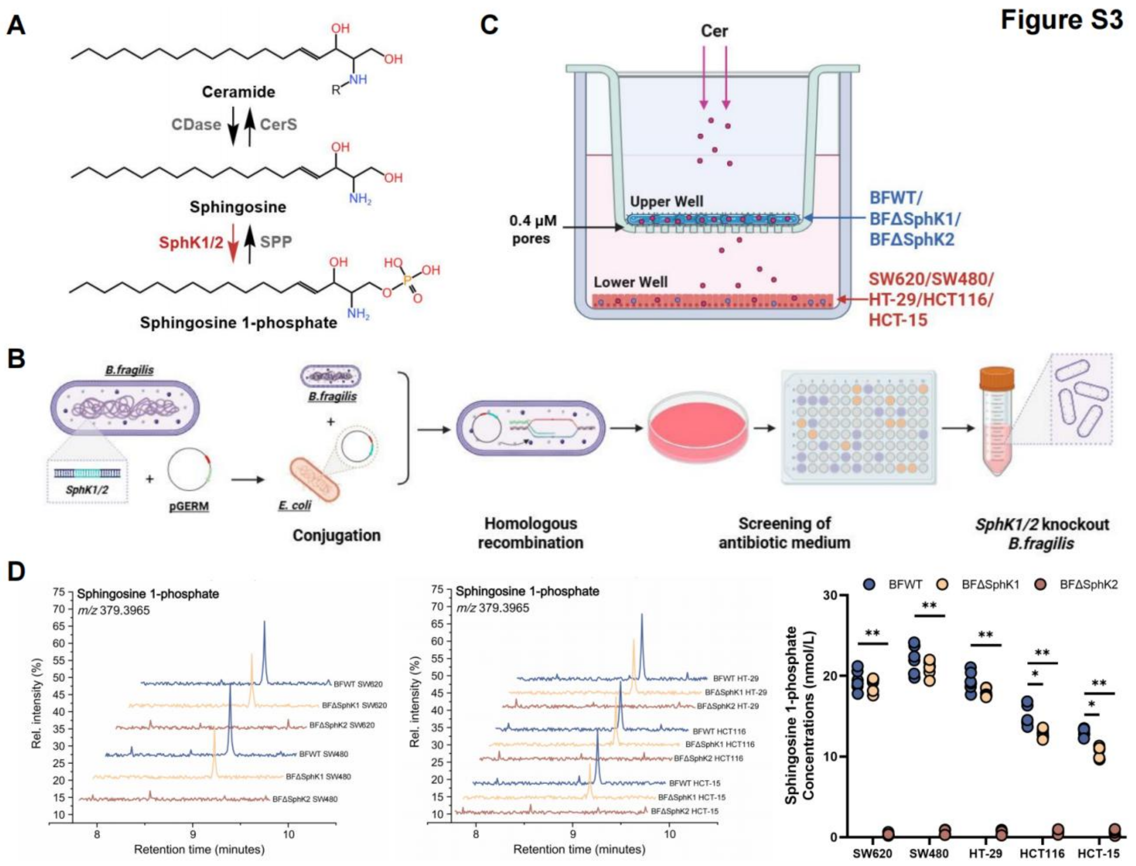

Figure S3. The synthesis of microbial S1P can penetrate into host CRC cells, related to Figure 3. (A) The key enzymes and substrates for the synthesis of microbial S1P from sphingolipid *de novo* synthesis pathway. (B) Schematic diagram illustrating the workflow for *SphK1/2* gene deletion in ETBF, which was created by Biorender. (C) Illustration of the transwell co-culture system, showcasing the transfer of S1P derivatives from ETBF or *E. coli* (upper well) to SW620, SW480, HT-29, HCT116 and HCT-15 cell lines (lower well). (D) Liquid chromatography-tandem mass spectrometry (LC-MS/MS) was utilized to identify and quantify S1P in lower well (left panel and middle panel). The curves of left and middle panels are representative images of each group. Data of right panel are presented as curves mean ± *SD*, **P* < 0.05, ***P* < 0.01 (Student's *t*-tests).

Figure S4

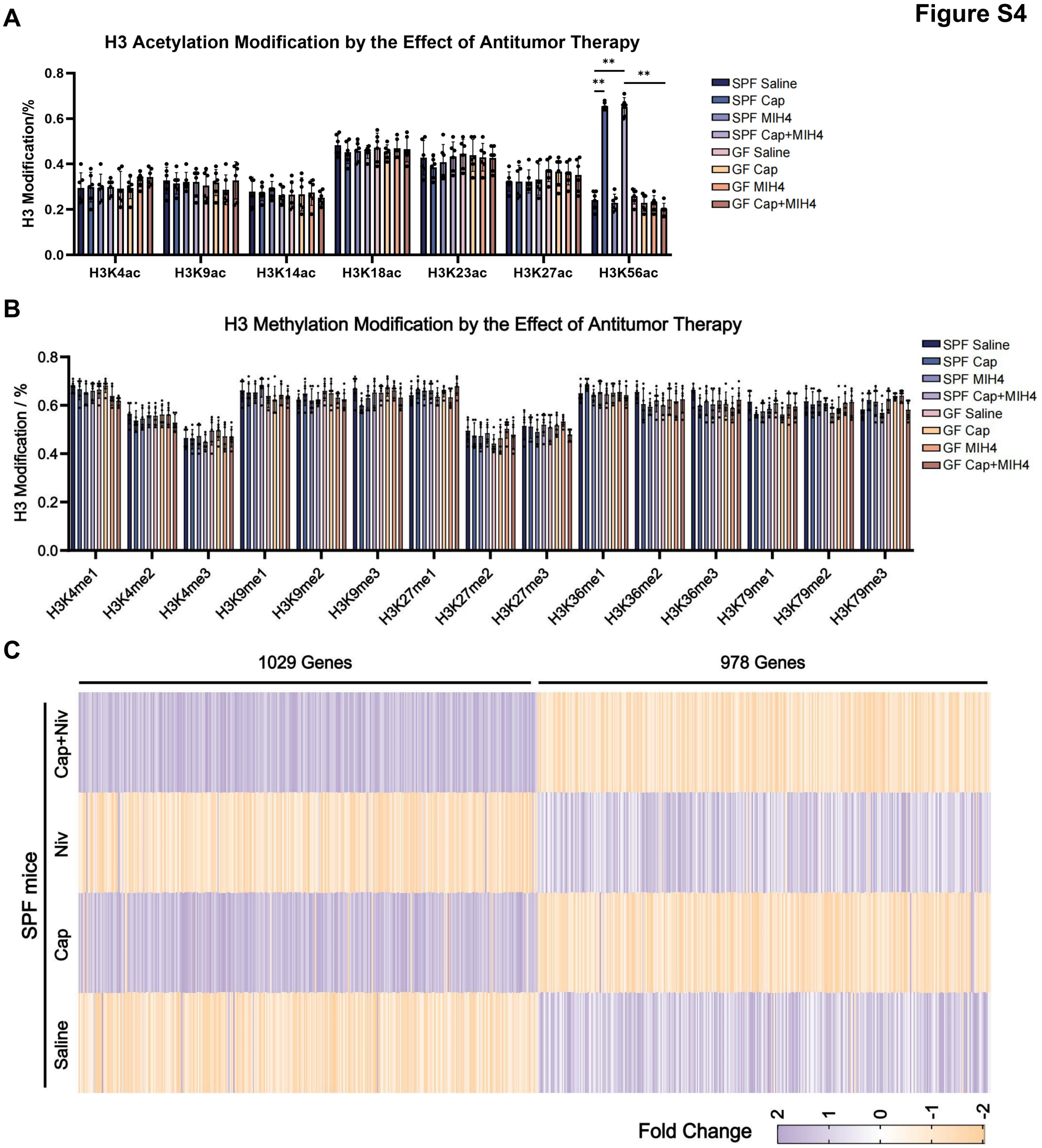

Figure S4. S1P originated from intratumoral microbiota affected histone acetylation by specifically binding to HDAC1, related to Figure 3. (A) Genome-wide screening assay analyzed the levels of acylation sites of H3 in the nucleus of colorectal epithelial cells isolated from SPF and GF mice. (B) Genome-wide screening assay analyzed the levels of methylation sites of H3 in the nucleus of colorectal epithelial cells isolated from SPF and GF mice. (C) The heat-map showed the differential expressed genes in CRC tissues of SPF or GF treated with Saline, Capecitabine, MIH4, or their combination.

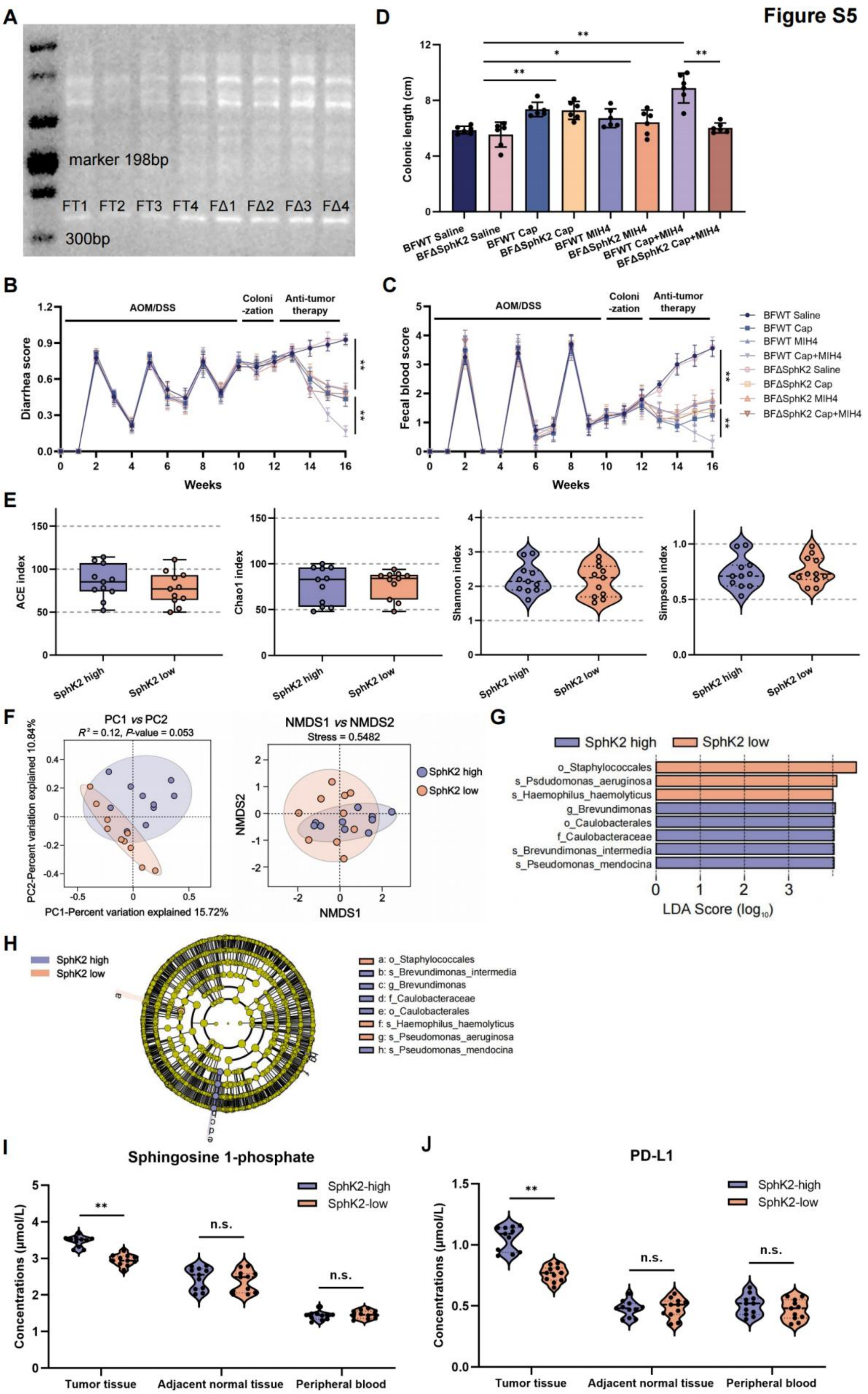

Figure S5
A
marker 198bp
FT1 FT2 FT3 FT4 FΔ1 FΔ2 FΔ3 FΔ4
300bp
D
Colonic length (cm)
BFWT Saline
BFΔSphK2 Saline
BFWT Cap
BFΔSphK2 Cap
BFWT MIH4
BFΔSphK2 MIH4
BFWT Cap+MIH4
BFΔSphK2 Cap+MIH4
B
AOM/DSS
Coloni -zation
Anti-tumor therapy
Diarrhea score
Weeks
C
Fecal blood score
BFWT Saline
BFWT Cap
BFWT MIH4
BFWT Cap+MIH4
BFΔSphK2 Saline
BFΔSphK2 Cap
BFΔSphK2 MIH4
BFΔSphK2 Cap+MIH4
E
ACE index
Chao1 index
Shannon index
Simpson index
SphK2 high
SphK2 low
F
PC1 vs PC2
R^2 = 0.12, P-value = 0.053
PC2-Percent variation explained 10.84%
PC1-Percent variation explained 15.72%
NMDS1 vs NMDS2
Stress = 0.5482
NMDS2
NMDS1
SphK2 high
SphK2 low
G
SphK2 high
SphK2 low
o_Staphylococcales
s_Psdudomonas_aeruginosa
s_Haemophilus_haemolyticus
g_Brevundimonas
o_Caulobacterales
f_Caulobacteraceae
s_Brevundimonas_intermedia
s_Pseudomonas_mendocina
LDA Score (log_{10})
H
SphK2 high
SphK2 low
a: o_Staphylococcales
b: s_Brevundimonas_intermedia
c: g_Brevundimonas
d: f_Caulobacteraceae
e: o_Caulobacterales
f: s_Haemophilus_haemolyticus
g: s_Pseudomonas_aeruginosa
h: s_Pseudomonas_mendocina
I
Sphingosine 1-phosphate
Concentrations (μmol/L)
SphK2-high
SphK2-low
n.s.
Tumor tissue
Adjacent normal tissue
Peripheral blood
J
PD-L1
Concentrations (μmol/L)
SphK2-high
SphK2-low
n.s.
Tumor tissue
Adjacent normal tissue
Peripheral blood

Figure S5. SphK2 activity of ETBF regulates the anti-tumor efficacy of Capecitabine-MIH4 combination treatment, related to Figs. 3 and 4. (A) Assessment of colonization fitness of ETBF wild-type (BFWT; FT1-4), and ETBF SphK2-knockout (BFΔSphK2; FΔ1-4) in GF mice. (B and C) Diarrhea score (B) and fecal blood score (C) of SPF and GF mice in each group were evaluated during the process of AOM/DSS induction, ETBF colonization and anti-tumor therapy. Data are means ± *SD*, $^{**}P < 0.01$ (Student's *t*-tests). (D) Colon lengths were measured in GF^{BFWT} and $GF^{BF\Delta SphK2}$ mice after the above 16-week of treatments. Data are means ± *SD*, $^{*}P < 0.05$, $^{**}P < 0.01$ (Student's *t*-tests). (E) α-diversity of the intratumoral microbiota between individuals with high SphK2 activity (SphK2-high) and low SphK2 activity (SphK2-low) treated with Capecitabine and MIH4 combination therapy, as indicated by the ACE, Chao1, Shannon and Simpson indexes. (F) β-diversity of the intratumoral microbiota between SphK2-high and SphK2-low individuals treated with Capecitabine-MIH4 combination therapy, as indicated by the scores of PCA and NMDS. (G and H) Taxonomic cladogram (G) and histogram (H) generated from LEfSe analysis of 16S rRNA gene sequences between SphK2-high and SphK2-low individuals treated with Capecitabine-MIH4 combination therapy. Each circle's size is proportional to the taxon's abundance. Only LDA scores > 4 are shown. (I) Quantification of intratumoral S1P concentrations, adjacent and peripheral tissues of patients with high or low microbial SphK2 activity, *n.s.* > 0.05, $^{**}P < 0.01$ (Student's *t*-tests). (J) Comparisons of intratumoral PD-L1 levels across CRC patients by WB assay. Data are means ± *SD*, n.s. > 0.05, $^{**}P < 0.01$ (Student's *t*-tests).

Figure S6

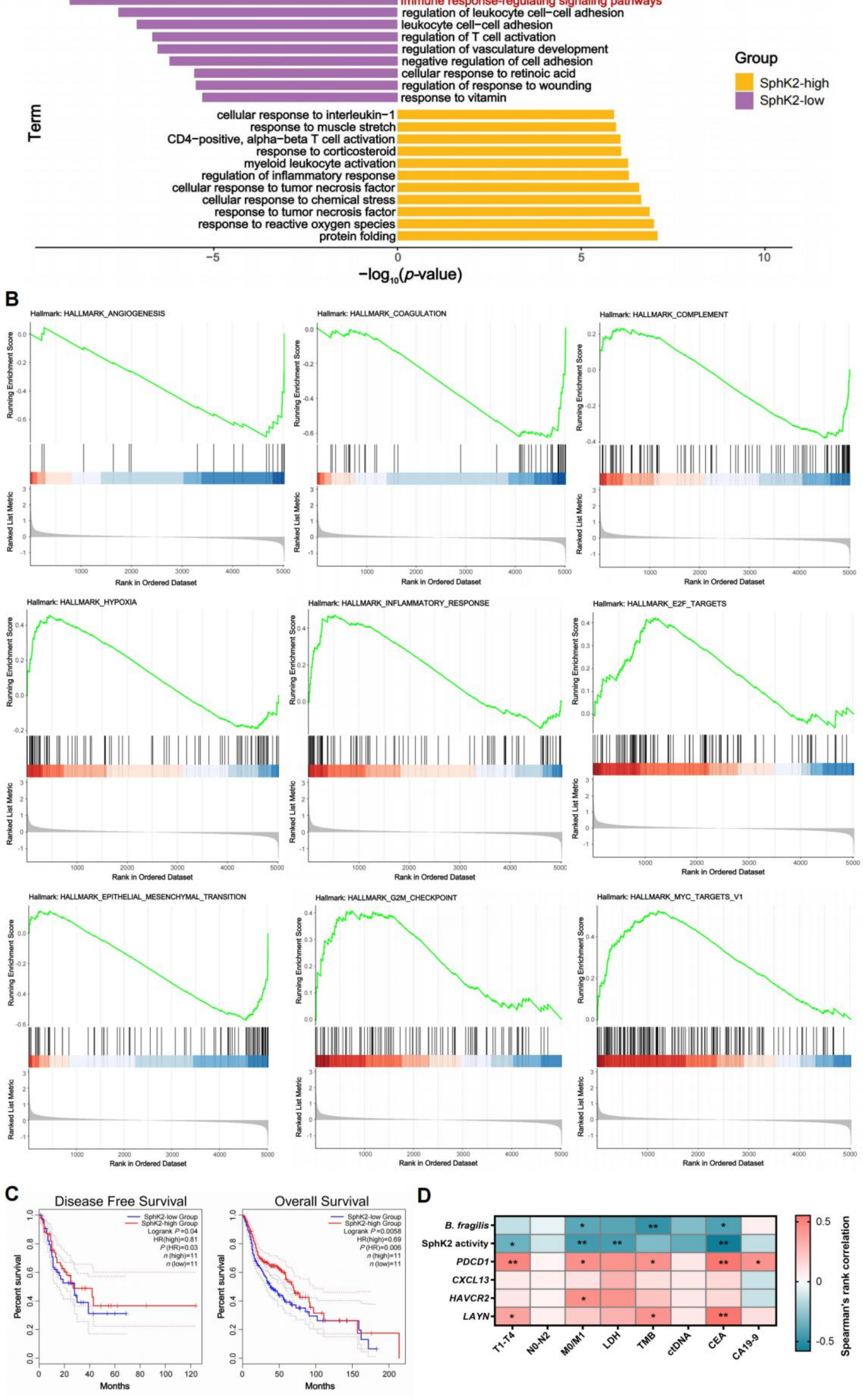
A
Immune response-regulating signaling pathways
regulation of leukocyte cell−cell adhesion
leukocyte cell−cell adhesion
regulation of T cell activation
regulation of vasculature development
negative regulation of cell adhesion
cellular response to retinoic acid
regulation of response to wounding
response to vitamin
cellular response to interleukin−1
response to muscle stretch
CD4−positive, alpha−beta T cell activation
response to corticosteroid
myeloid leukocyte activation
regulation of inflammatory response
cellular response to tumor necrosis factor
cellular response to chemical stress
response to tumor necrosis factor
response to reactive oxygen species
protein folding
Term
Group
SphK2-high
SphK2-low
−log10(p-value)
B
Hallmark: HALLMARK_ANGIOGENESIS
Hallmark: HALLMARK_COAGULATION
Hallmark: HALLMARK_COMPLEMENT
Hallmark: HALLMARK_HYPOXIA
Hallmark: HALLMARK_INFLAMMATORY_RESPONSE
Hallmark: HALLMARK_E2F_TARGETS
Hallmark: HALLMARK_EPITHELIAL_MESENCHYMAL_TRANSITION
Hallmark: HALLMARK_G2M_CHECKPOINT
Hallmark: HALLMARK_MYC_TARGETS_V1
Running Enrichment Score
Ranked List Metric
Rank in Ordered Dataset
C
Disease Free Survival
SphK2-low Group
SphK2-high Group
Logrank P=0.04
HR(high)=0.81
P (HR)=0.03
n (high)=11
n (low)=11
Overall Survival
SphK2-low Group
SphK2-high Group
Logrank P=0.0058
HR(high)=0.69
P (HR)=0.006
n (high)=11
n (low)=11
Percent survival
Months
D
B. fragilis
SphK2 activity
PDCD1
CXCL13
HAVCR2
LAYN
T1-T4
N0-N2
M0/M1
LDH
TMB
ctDNA
CEA
CA19-9
Spearman's rank correlation

Figure S6. The SphK2 high activity cohort benefited more from Capecitabine-Nivolumab combination therapy, related to Figure 6. (A) GO analysis analyzes pathway enrichment after the treatment of Capecitabine-Nivolumab combination. Microbial SphK2-high expression group is indicated in purple; pathways associated with inflammation with low microbial SphK2 expression are indicated in yellow. (B) Running Enrichment Score curve of Gene Set Enrichment Analysis (GSEA). **Hallmark_Angiogenesis:** core genes related to angiogenesis, involved in the formation of new blood vessels during processes such as tumor growth and tissue repair. **Hallmark_Coagulation:** core genes related to the coagulation cascade, involving processes such as blood coagulation, thrombosis, and inflammatory response. **Hallmark_Complement:** enrichment correlates with diminished life metrics (*e.g.*, lower survival, impaired health). **Hallmark_Hypoxia:** enrichment suggests low oxygen state in tumor micro-environment. **Hallmark_Inflammatory Response:** genes involved in immune and inflammatory processes. **Hallmark_Epithelial Mesenchymal Transition:** enrichment links to worsened prognosis (13% survival decline, aggressive disease). **Hallmark_Checkpoint:** This gene set's enrichment shows tight linkage to cellular proliferation. **Hallmark_MYC_Targets_v1:** the enrichment of this gene set suggests abnormal activation of the MYC pathway in tumor samples. The X-axis represents the position of genes in the sorted data-set, and the Running Enrichment Score (ES) is a dynamically calculated statistic. The peak of the curve in the chart reflects the degree of enrichment. FDR q-value < 0.05. (C) The Kaplan-Meier overall survival curve in patients with CRC, grouped by different expression levels of microbial SphK2. Patients separated by high (n = 11) or low-level (n = 11) microbial SphK2 signature exhibit different disease-free survival and overall survival (P < 0.05). (D) Microbial SphK2 activity, relative abundance of intratumoral ETBF, expressions of *PDCD1*, *CXCL13*, *HAVCR2*, *LAYN* to correlate with CRC indicators.

Abbreviation List

CRC, colorectal cancer; GF, germ-free; SPF, specific-pathogen-free; S1P, sphingosine-1 -phosphate; ETBF, enterotoxigenic *Bacteroides fragilis*; *E. coli*, *Escherichia coli*; SphK1/2, sphingosine kinase 1/2; FDA, Food and Drug Administration; PD-1, programmed death receptor-1; PD-L1, programmed death ligand-1; dMMR/MSI-H, deficient/microsatellite instability-high; mCRC, metastatic CRC; Cap, Capecitabine; Niv, Nivolumab; AOM, azoxymethane; DSS, dextran sulfate sodium; SLs, sphingolipids; BFΔSphK1/2, mutant ETBF strains with inactivated SphK1/2; BFWT, wild-type ETBF; UPLC-ESI-QTOF/MS, ultra performance liquid chromatography coupled time-of-flight mass spectrometry; Co-IP, co-immunoprecipitation; GST, glutathione S-transferase; RNA-seq, RNA sequencing; DEGs, differentially expressed genes; ChIP-seq, chromatin immunoprecipitation sequencing; GF^{BFWT}, GF mice colonized with BFWT; $GF^{BF\Delta SphK2}$, GF mice colonized with BFΔSphK2; scRNA-Seq, single-cell RNA sequencing; ILCs, innate lymphoid cells; Robs/exp, ratio of observed to expected cell numbers; Tex, exhausted T cells; DFS, disease-free survival; OS, overall survival; qPCR, quantitative PCR; FDR, false discovery rate; RT, retention time; *m/z*, mass-to-charge ratio; OTUs, operational taxonomic units; PCA, principal component analysis; PCoA, principal coordinates analysis; LEfSe, Linear Discriminant Analysis Effect Size; FBS, fetal bovine serum; PBMCs, peripheral blood mononuclear cells; PBS, phosphate-buffered saline; FACS, fluorescence-activated cell sorting; PFA, paraformaldehyde; UMI, unique molecular identifiers; HVGs, highly variable genes; *Ro/e*, observed-to-expected; ANOVA, analysis of variance.

Table S1. Primers for RT-qPCR assay

Genes/bacteria	Forward (5'-3') primer	Reward (5'-3') primer
27F	GTTTGATCCTGGCTCAG	/
1492R	/	CGGCTACCTTGTTACGAC
B. fragilis	TCRGGAAGAAAGCTTG CT	ACACGTATCCAACCTGCCC TTTACTCG
Gapdh	TGACGTGCCGCCTGGA GAAA	AGTGTAGCCCAAGATGCCC TTCAG

Table S2. Sphingolipids detected by LC-MS

**Compound Name**	**Precursor Ion**	**Product Ion**	**Fragmentation (V)**	**Collision Energy (V)**	**Ret Time (min)**	**Polarity**
S1P(d17:1)	248.4	230.4	125	10	0.753	Positive
S1P(d18:1)	262.5	244.5	151	8	0.836	Positive
Sa (d17:0)	226.5	208.5	177	8	0.71	Positive
Sa (d18:0)	240.3	222.3	170	8	0.764	Positive
Sa (d18:1)	238.7	220.7	146	10	0.773	Positive
Sa (m17:0)	210.8	192.8	138	16	0.693	Positive
DHCer(d18:0/d14:0)	340.9	322.9	111	12	1.035	Positive
DHCer(d18:0/d16:0)	368.6	350.6	133	14	1.175	Positive
DHCer(d18:0/d16:1)	366.6	348.6	134	14	1.284	Positive
DHCer(d18:0/d18:0)	396.4	378.4	161	16	1.729	Positive
DHCer(d18:0/d18:1)	394.5	376.5	158	14	1.845	Positive
DHCer(d18:0/d20:0)	424.5	406.5	118	18	1.719	Positive
DHCer(d18:0/d20:1)	422.7	404.7	108	18	1.827	Positive
DHCer(d18:0/d22:0)	454.3	436.3	172	20	1.988	Positive
DHCer(d18:0/d22:1)	452.5	434.5	101	24	2.074	Positive

DHCer(d18:0/d24:0)	482.8	464.8	165	24	2.836	Positive
DHCer(d18:0/d24:1)	480.8	462.8	109	24	3.574	Positive
Cer(d18:1/d14:0)	338.7	320.7	110	10	0.945	Positive
Cer(d18:1/d16:0)	366.5	348.5	168	12	1.034	Positive
Cer(d18:0/16:1)	364.3	346.3	161	12	1.173	Positive
Cer(d18:1/18:0)	394.1	376.1	162	16	1.682	Positive
Cer(d18:0/18:1)	392.2	374.2	164	16	1.748	Positive
Cer(d18:0/20:0)	422.4	404.4	164	14	1.659	Positive
Cer(d18:1/20:1)	420.5	402.5	125	18	1.727	Positive
Cer(d18:1/d22:0)	452.3	434.4	120	16	1.883	Positive
Cer(d18:0/22:1)	450.7	432.7	137	16	1.922	Positive
Cer(d18:1/24:0)	480.3	462.3	169	18	2.747	Positive
Cer(d18:0/24:1)	478.6	460.6	119	20	3.438	Positive
Cer(d18:0/26:0)	508.5	490.5	158	18	3.554	Positive
Cer(d18:0/26:1)	506.8	488.8	159	18	3.827	Positive
So(d17:1)	170.3	162.3	105	8	0.577	Positive
Sph(d17:1)	306.4	298.4	197	12	0.969	Positive
Sph(d18:1)	322.7	304.7	110	12	1.058	Positive
